# Fossil-calibrated molecular phylogeny of atlantid heteropods (Gastropoda, Pterotracheoidea)

**DOI:** 10.1186/s12862-020-01682-9

**Published:** 2020-09-21

**Authors:** Deborah Wall-Palmer, Arie W. Janssen, Erica Goetze, Le Qin Choo, Lisette Mekkes, Katja T. C. A. Peijnenburg

**Affiliations:** 1 Plankton Diversity and Evolution, Nauralis Biodiversity Center, Leiden, The Netherlands; 2 Department of Oceanography, University of Hawai’i at Mānoa, Honolulu, USA; 3 Institute for Biodiversity and Ecosystem Dynamics (IBED), University of Amsterdam, Amsterdam, The Netherlands

**Keywords:** Atlantidae, Planktonic gastropods, Cytochrome *c* oxidase subunit 1, 28S and 18S ribosomal rRNA, Ocean change, Rapid diversification

## Abstract

**Background:**

The aragonite shelled, planktonic gastropod family Atlantidae (shelled heteropods) is likely to be one of the first groups to be impacted by imminent ocean changes, including ocean warming and ocean acidification. With a fossil record spanning at least 100 Ma, atlantids have experienced and survived global-scale ocean changes and extinction events in the past. However, the diversification patterns and tempo of evolution in this family are largely unknown.

**Results:**

Based on a concatenated maximum likelihood phylogeny of three genes (cytochrome *c* oxidase subunit 1 mitochondrial DNA, 28S and 18S ribosomal rRNA) we show that the three extant genera of the family Atlantidae, *Atlanta, Protatlanta* and *Oxygyrus,* form monophyletic groups. The genus *Atlanta* is split into two groups, one exhibiting smaller, well ornamented shells, and the other having larger, less ornamented shells. The fossil record, in combination with a fossil-calibrated phylogeny, suggests that large scale atlantid extinction was accompanied by considerable and rapid diversification over the last 25 Ma, potentially driven by vicariance events.

**Conclusions:**

Now confronted with a rapidly changing modern ocean, the ability of atlantids to survive past global change crises gives some optimism that they may be able to persist through the Anthropocene.

## Background

**Fig. 1:**
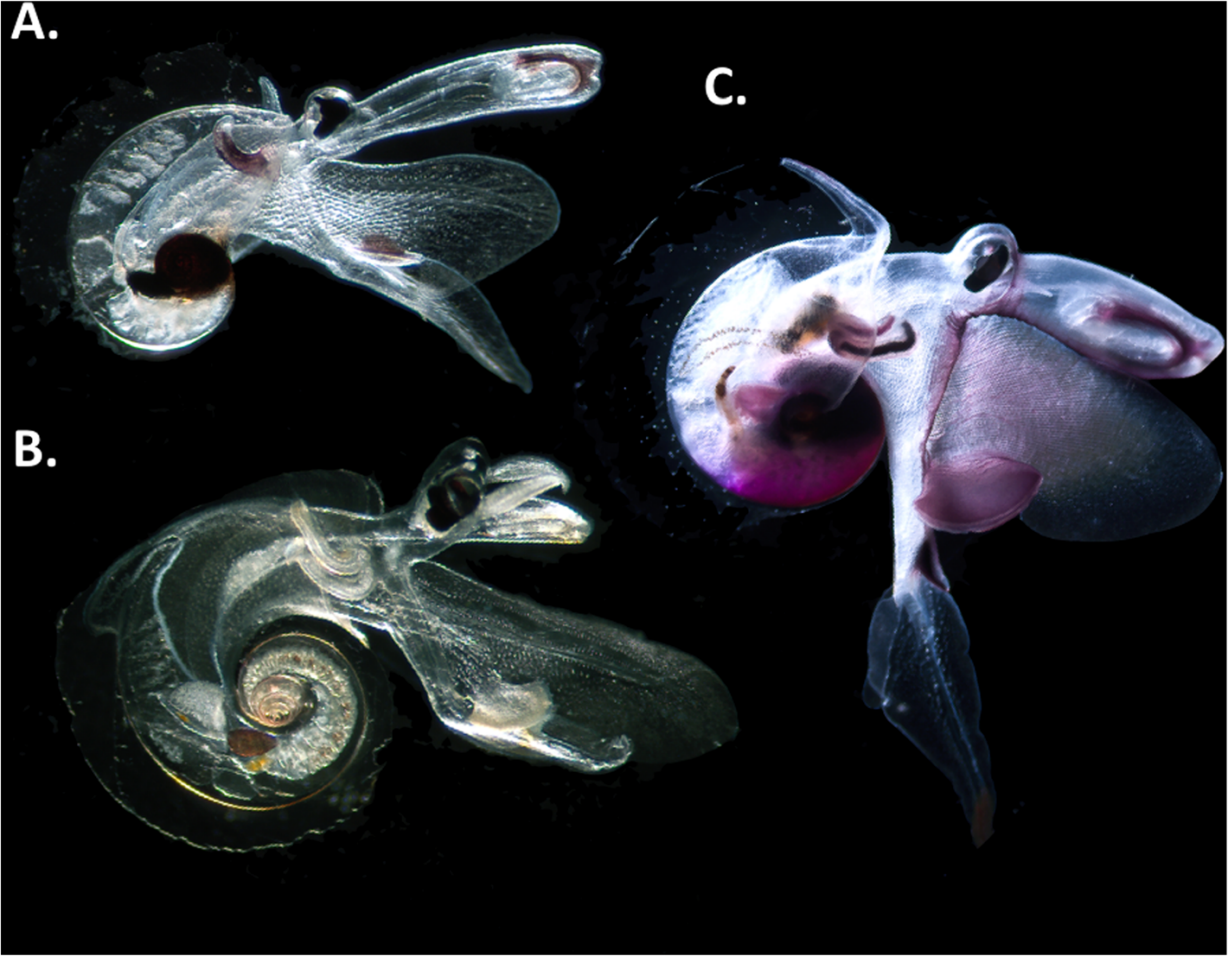
Adult representatives of the three extant Atlantidae genera. **a** *Protatlanta souleyeti,* **b** *Atlanta gibbosa,* **c** *Oxygyrus inflatus.* All specimens were collected and photographed during the AMT27 cruise from the Atlantic Ocean

The Atlantidae is a family of small (< 14 mm) marine predatory gastropods with a holoplanktonic mode of life (Fig. 1). Atlantids fall within the superfamily Pterotracheoidea, known commonly as heteropods, or sea elephants. Unlike the other two heteropod families (Carinariidae and Pterotracheidae), all three genera of the Atlantidae (*Atlanta, Protatlanta* and *Oxygyrus*) have thin-walled laterally compressed aragonite shells that are broadened with a keel. A modified foot serves as a primary swimming fin, and the broad shell is used as a secondary swimming fin [1]. Together, the fin and the shell generate rapid and directed movement for prey capture and predator evasion. Atlantids are able to fully withdraw into their shell and seal the aperture with an operculum. They also have well developed eyes, a sucker on their fin for securing prey, and a proboscis, or trunk, which is used for reaching into the shells of prey, such as shelled pteropods [2–4]. It is clear that atlantids have remarkable and derived adaptations for a holoplanktonic lifestyle, however, their evolutionary history is largely unknown. Until now, hypotheses about the evolution of atlantids (and heteropods in general) have relied upon a fossil record with vast gaps [5], due to a combination of the loss of delicate aragonite shells during diagenetic processes, creating genuine gaps in the fossil record, and a lack of research on fossil heteropods as a whole, creating knowledge gaps. Despite these gaps, the evolutionary history of this group is of interest in understanding how these delicate aragonite shelled plankton, and presently the only aragonite shelled predatory holoplankton, have fared through past climate change and ocean acidification events, since the Early Cretaceous. The morphologically similar aragonite shelled pteropods are known to have survived through both the Cretaceous-Paleogene (KPg or KT) extinction event and the Paleocene Eocene Thermal Maximum (PETM), which were both times of extreme climate change and the closest analogues to predicted ocean changes [6–8].

The thin-walled aragonite shells of the atlantids, and their habitat in the upper ocean imply that they are likely to be sensitive to ocean acidification and ocean warming, in a similar way to the shelled pteropods [9]. The only study addressing the effects of ocean acidification on atlantids found negative effects of reduced ocean pH on shell growth and the down-regulation of biomineralisation and growth genes [10]. Relatively recent local extinctions have been reported for several atlantid species. *Atlanta plana* Richter, 1972 and *Atlanta turriculata* d’Orbigny, 1836 are not found in the modern Atlantic Ocean [11], however, fossils of both species have been found in Late Pleistocene sediments of the Caribbean Sea [12], and *A. plana* has also been found in Pliocene rocks of southern France and southern Spain [13, 14]. These records suggest the local extinction of *A. turriculata* at around 16 thousand years (ka), and *A. plana* in the last 3.5–1 ka. *Protatlanta sculpta* Issel, 1911 is currently only known from the Atlantic Ocean, but was present in Late Pleistocene sediments of the Indian Ocean 24–16 ka ago (D. Wall-Palmer personal observation) and in Pliocene rocks of Pangasinan, Philippines [15]. Most of these local extinctions have occurred within the warming period since the Last Glacial Maximum, and may reflect sensitivity to a changing ocean.

**Fig. 2:**
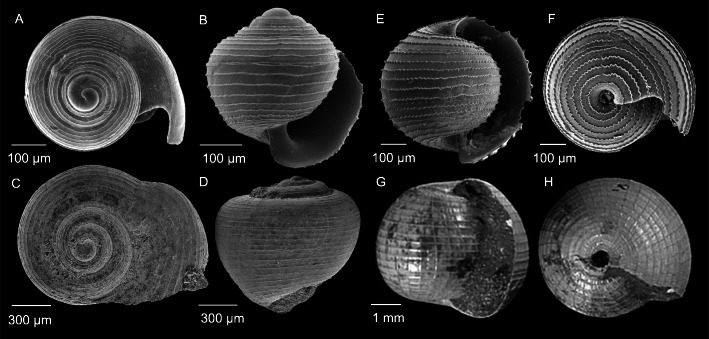
Comparisons in shell morphology between the oldest fossil heteropods and larval shells of extant members of the Atlantidae. **a** Juvenile *Atlanta selvagensis* collected live from the Atlantic Ocean in 2010, **b** Juvenile *A. selvagensis,* a recent fossil from Caribbean sediments, **c**–**d** The oldest potential heteropod, *Coelodiscus minutus* from the Early-Middle Jurassic (190.8–170.3 Ma). Specimens BSPG 2008 XXIX 42c and BSPG 2008 XXIX 56f, images from Teichert and Nützel [16], **e**–**f** Juvenile *Oxygyrus inflatus* collected live from the Atlantic Ocean in 2010, **g**–**h** The oldest potential Atlantidae, *Belerophina minuta* from the Early Cretaceous (~ 113–100.5 Ma). *Bellerophina* images courtesy of Steven Tracey [22]

Holoplanktonic gastropods are thought to have evolved from benthic gastropods with planktotrophic larvae, with likely progression to remain planktonic in response to increasing hostility of the sea floor, including anoxic bottom conditions [16] and/or an increase in benthic shell destroying predators [17]. Holoplanktonic gastropods first appear in the Jurassic, coinciding with the Early Jurassic Anoxia Event [18]. Amongst the earliest holoplanktonic gastropods are several potential heteropod genera including *Coelodiscus, Freboldia* and *Tatediscus* [16, 19, 20]. *Coelodiscus minutus* (Schübler, 1833) [16, 21] from the Pliensbachian–Aalenian of the Early–Middle Jurassic (190.8–170.3 Ma) is the earliest known holoplanktonic caenogastropod and probable heteropod. *Coelodiscus minutus* has a shell morphology remarkably similar to larval atlantid shells of the genus *Atlanta* (Fig. 2a-d)*.* It is thought that *C. minutus* does represent an early heteropod [19], but only two ontogenetic stages can be identified from abundant fossil material (compared to three in modern heteropods), and therefore it is not a member of any of the extant families [16]. The planktotrophic larval stage of *C. minutus* probably became the adult stage when transitioning to a holoplanktonic mode of life, and the adult shell of the modern atlantid heteropods developed later [16].

The oldest potential member of the family Atlantidae does not appear in the fossil record until ~ 57 Ma later, in the Early Cretaceous. *Bellerophina minuta* (Sowerby, 1812) [22, 23], found in the Albian (~ 113–100.5 Ma), has an involute, more rounded shell morphology with clear ornamentation that is similar to larval shells of the extant atlantid genus *Oxygyrus* (Fig. 2e-h)*.* Destombes [24] considered the relationship between *B. minuta* and the genus *Oxygyrus* to be unclear, due to differences in size and incompleteness of the aperture in *B. minuta* fossils. He therefore placed *B. minuta* into a separate family, Bellerophinidae, in which an older genus *Freboldia* (163.5–157.3 Ma) is now also placed [18]. However, the relationship between *Oxygyrus* and *B. minuta* remains enigmatic [22], and here we consider *B. minuta* to belong within the family Atlantidae, having a shell morphology very similar to the extant genus *Oxygyrus* [22]. Indeed, the World Register of Marine Species places the genus *Bellerophina* within the Family Atlantidae. *Freboldia fluitans* Nützel & Schneider, 2016 (163.5–157.3 Ma) is thought to be holoplanktonic and its shell morphology is involute and quite rounded in shape, however, unlike *B. minuta,* the shell surface has little or no ornamentation, and the coiling direction of *F. fluitans* cannot be determined with certainty [18].

Through the Late Cretaceous, Paleocene and Eocene there are no known atlantid fossils, creating a gap in the record of ~ 73 Ma. The recent fossil record of the Atlantidae, extending to the Piacenzian (3.6–0 Ma) is relatively well known [5]. However, from the Piacenzian to the Chattian of the Oligocene (~ 27.82–3.6 Ma) [25], diversity is much lower with only five atlantid species, and two not determined to species level. There are eight atlantid species known to have become extinct during the Miocene and the Pliocene [5], and one extinct genus, *Atlantidea* [26].

Although heteropods have almost certainly been alive for the last ~ 190 Ma, nothing is known from these large 57 Ma and 73 Ma gaps in the fossil record, and so the evolutionary diversification patterns and timing of this group are unclear. It has been hypothesised that within the family Atlantidae, the genus *Atlanta,* with an entirely aragonite shell, is the earliest diverged, and that the genus *Oxygyrus,* with a shell composed of both aragonite and conchiolin (probably to improve buoyancy), is the latest to diverge [2, 27–30]. However, the shell of the Early Cretaceous *B. minuta* is morphologically most similar to *Oxygyrus* (Fig. 2), contradicting this hypothesised evolutionary history of the atlantids.

Only a single large-scale study has previously explored the molecular phylogeny of the atlantid heteropods [11]. Wall-Palmer et al. [11] revealed considerable hidden diversity using a global dataset of mitochondrial cytochrome *c* oxidase subunit 1 (CO1) sequences from specimens of all known atlantid morphospecies. However, deeper genus level relationships were not resolved in this study, and it provided few clues about the longer-term evolutionary history of the family. In the present study, an extended CO1 dataset is used in combination with two nuclear genes, 28S and 18S, to produce a more complete molecular phylogeny with which to compare previous morphology based hypotheses of atlantid evolution. Through a fossil-calibration of this phylogeny, the likely timing of diversification reveals the persistence of this successful group of holoplankton through past ocean changes.

## Results and discussion

### Phylogeny of the Atlantidae

**Fig. 3:**
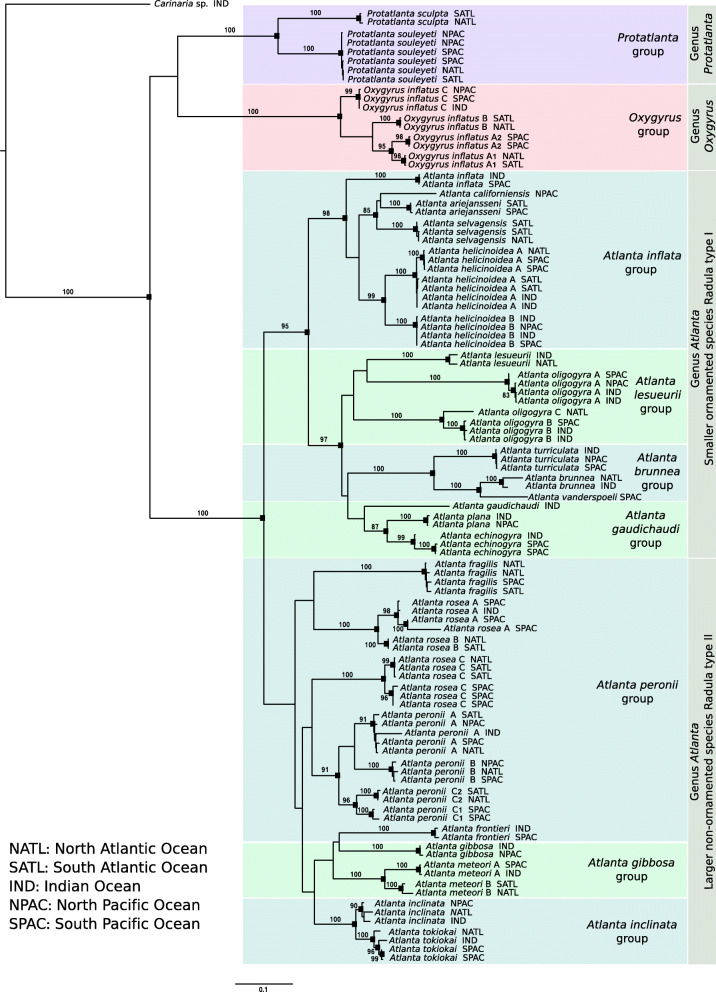
Maximum likelihood phylogeny of the family Atlantidae based on a concatenated dataset of cytochrome *c* oxidase subunit 1 mitochondrial DNA (CO1), 28S and 18S ribosomal rRNA (total 2472 bp). Black squares represent bootstrap support > 80%. Species groups based on morphology are highlighted with coloured boxes (see Table 1)

Phylogenetic analysis of a concatenated (3-gene) alignment of CO1, 28S and 18S recovered species and genera, with node supports of > 80% (100% for most) at most levels of the atlantid tree (Fig. 3). For the first time, it can be demonstrated that all three atlantid genera are monophyletic with 100% bootstrap support, however, relationships between the genera remain inconclusive. Maximum likelihood analyses of individual genes recovered species and genera with varying levels of success (Figs. S1, S2, S3). While all 34 previously known putative atlantid species [11] (including all 24 morphospecies) were resolved by the CO1 phylogenetic tree (supports of > 80, 100% for most), two additional putative species were also detected and verified by ABGD analysis (one within *A. peronii* C, and one within *O. inflatus* A). This brings the total number of putative species to 36, of which 24 are described as morphospecies and 12 are undescribed putative species. However, the deeper relationships between atlantid genera were not supported within the CO1 phylogenetic tree (< 60%, Fig. S1). Conversely, 28S and 18S trees did not always resolve species-level relationships. The 28S phylogeny supported 13 of the described morphospecies and a further seven of the undescribed putative species, whereas the 18S phylogeny supported only four described morphospecies and four undescribed putative species. The 28S and 18S trees did, however, provide moderate support for relationships among the atlantid genera (> 60%, Figs. S2 and S3, respectively).

Atlantids have long been divided into groups of closely related species (Table 1) based on morphological characters. The 3-gene maximum likelihood (ML) phylogeny largely supports these morphology based species groups (Table 1), with only the *Atlanta peronii* Lesueur, 1817*, Atlanta gibbosa* Souleyet, 1852 and *Atlanta lesueurii* Gray, 1850 groups not resolved (Fig. 3). The *A. lesueurii* group is monophyletic, but not supported (bootstrap support 37%). The *A. peronii* and *A. gibbosa* groups are not monophyletic due to the position of *A. frontieri* Richter, 1993, which falls within the *A. gibbosa* group (Fig. 3). As found in previous phylogenetic analysis of CO1 [11], several atlantid morphospecies contain one or more additional well-supported putative species (bootstrap support > 80%). Here we find that six morphospecies contain a total of 17 putative species. The majority of these putative species (11 of 17) show some degree of geographical separation, residing in different ocean basins, and seven of these putative species are also supported by the more slowly evolving, independently inherited nuclear markers, demonstrating that they are probably distinct species. However, further work in resolving the morphology and distributions of these new species would be necessary to describe and validate each one [32].

**Table 1:** A summary of the morphology based species groups, key morphological characters for each group [31], and the phylogenetic support obtained for each group in our analyses

Species group	Species	Radula type	Shell and keel composition	Morphological characters of this group	Is the group supported by the 3-gene phylogeny?
*Atlanta brunnea*	*Atlanta brunnea* Gray, 1850	I	Both aragonite	Small shell (< 2 mm) with a tall keel. Larval shell is tall, conical, covered with ornamentation and with a prominent carina slightly above mid-whorl height	Yes
	*Atlanta turriculata* d’Orbigny, 1836	I			
	*Atlanta vanderspoeli* Wall-Palmer, Hegmann & Peijnenburg, 2019	I			
*Atlanta inflata*	*Atlanta ariejansseni* Wall-Palmer, Burridge & Peijnenburg, 2016	I	Both aragonite	Small-medium sized shell (< 4 mm) with a short or tall keel. Larval shell is flattened, or low conical and either smooth, or ornamented with evenly spaced spiral ridges or punctae.	Yes
	*Atlanta californiensis* Seapy & Richter, 1993	I			
	*Atlanta helicinoidea (A and B)* Gray, 1850	I			
	*Atlanta inflata* Gray, 1850	I			
	*Atlanta selvagensis* de Vera & Seapy, 2006	I			
*Atlanta lesueurii*	*Atlanta lesueurii* Gray, 1850	I	Both aragonite	The larval shell is very small, with only 2.5 whorls.	Yes - species group together but node support is low (37%).
	*Atlanta oligogyra (A, B and C)* Tesch, 1906	I			
*Atlanta gaudichaudi*	*Atlanta echinogyra* Richter, 1972	I	Both aragonite	Small-medium shell (< 4 mm). Larval shell flattened to conical with varying numbers of spiral lines (only 1 in *A. gaudichaudi*).	Yes, but node support is moderate (75%).
	*Atlanta gaudichaudi* Gray, 1850	I			
	*Atlanta plana* Richter, 1972	I			
*Atlanta peronii*	*Atlanta fragilis* Richter, 1993	II	Both aragonite	Medium-large sized shell (< 10 mm). Larval shell is flattened or low conical with no ornamentation (except *A. frontieri*).	No - species do not group together. *A. frontieri* groups with *A. gibbosa* group.
	*Atlanta frontieri* Richter, 1993	II			
	*Atlanta peronii (A, B and C)* Lesueur, 1817	II			
	*Atlanta rosea (A, B and C)* Gray, 1850	II			
*Atlanta inclinata*	*Atlanta inclinata* Gray, 1850	II	Both aragonite	Large shell (< 6 mm) with a tall keel. Larval shell large, conical and globose, tilted relative to the adult shell. Larval shell covered with small tubercula.	Yes
	*Atlanta tokiokai* van der Spoel & Troost, 1972	II			
*Atlanta gibbosa*	*Atlanta gibbosa* Souleyet, 1852	II	Both aragonite	Medium shell (< 4 mm) with a tall keel that is very thin and transparent. Larval shell large, conical and globose, tilted relative to the adult shell. Larval shell smooth.	No - species group together with *A. frontieri.* Low node support (32%).
	*Atlanta meteori (A and B)* Richter, 1972	II			
*Oxygyrus*	*Oxygyrus inflatus (A, B and C)* Benson, 1835	I	Inner shell aragonite, outer shell and keel of conchiolin.	Large shell (< 14 mm) very rounded, almost spherical and involute with a tall conchiolin keel. Larval shell heavily ornamented with zig-zig spiral lines. The final whorl of the adult shell is partially composed of conchiolin.	Yes
*Protatlanta*	*Protatlanta sculpta* Issel, 1911	I	Aragonite shell, conchiolin keel	Small shell (< 2 mm) of aragonite with a tall conchiolin keel. Larval shell is low conical with 2.5–3 whorls.	Yes
	*Protatlanta souleyeti* (Smith, 1888)	I			

The 3-gene phylogeny demonstrates that the genus *Atlanta* can be split into two broad groups: one of smaller-shelled species generally with shell ornamentation, and one of larger-shelled species generally lacking shell ornamentation (Fig. 3). The smaller ornamented species form a well-supported monophyletic group containing the *Atlanta inflata* Gray, 1850*, A. brunnea* Gray, 1850*, A. gaudichaudi* Gray, 1850 and *A. lesueurii* species groups (95% supported). The larger and non-ornamented shelled species are grouped together, but have low node support (58% support). These two broad groups are also supported by radula type (Table 1), with the smaller ornamented group having a ‘type I radula’, in which the number of tooth rows continually increases because teeth are never cast off, and the larger non-ornamented group having a ‘type II radula’, with a set number of tooth rows per species because teeth are cast off the anterior end [33]. These results suggest that the genus *Atlanta* has followed two evolutionary routes.

Previous morphology based studies that focussed on the shell, eyes, radula and operculum [27–30], as well as chromosomal studies [34], also support a lineage of larger-shelled species generally lacking shell ornamentation. Richter [27–30] proposed an evolutionary route where shells became flatter, shell walls became thinner and the central spire became narrower and tilted over evolutionary time. The lineage of larger, non-ornamented species identified in the present study do exhibit these shell features. Richter proposed that this evolutionary path resulted from selective pressure to improve swimming efficiency by providing a broad flat shell to counteract the side-to-side swimming motion. This may partly be true, however, we now know that the shell is used as a secondary swimming fin by *A. selvagensis* de Vera & Seapy, 2006, and likely by all atlantid species [1] so a broadening of the shell may permit faster swimming speeds.

Richter [27–30] proposed a second evolutionary route within the genus *Atlanta* that involves the reduction of shell mass, where shells gradually become composed of less aragonite and more conchiolin. This route would involve the direct evolution from the ornamented members of the genus *Atlanta* (fully aragonite shell, Fig. 1b), to *Protatlanta* (aragonite shell, conchiolin keel, Fig. 1a) and terminating in *Oxygyrus* (shell largely composed of conchiolin, Fig. 1c). The monophyletic group of smaller, ornamented *Atlanta* species identified in the present study does share the same radula ‘type I’ with *Protatlanta* and *Oxygyrus.* However, the relationships between the three Atlantidae genera are not resolved, and therefore, this evolutionary hypothesis cannot be tested. A broader dataset including information from the other two heteropod families and more genetic information is needed to resolve and inform these three deepest nodes within the atlantid phylogeny.

### Gaps in the fossil record

**Fig. 4:**
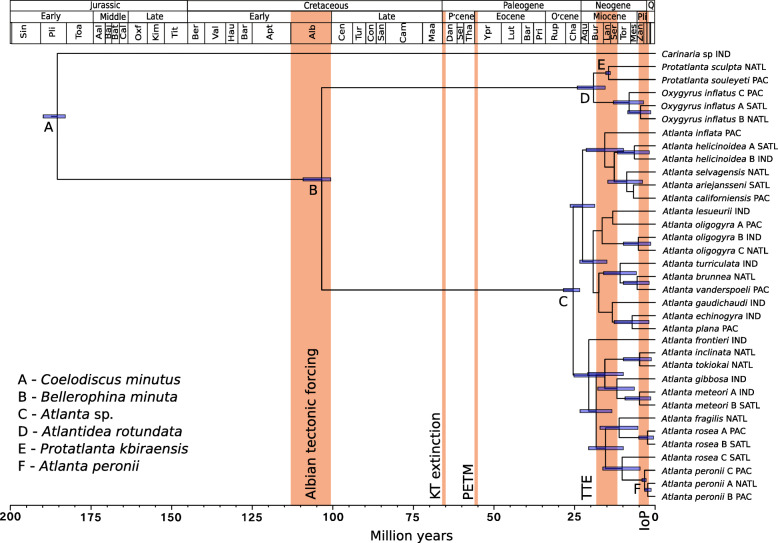
The fossil-calibrated phylogeny of the family Atlantidae. Error bars shown in purple (95%) are presented for putative species with posterior probabilities ≥85%. Calibration fossils are indicated with letters A-F (see legend and Table 3). Major geological events, including the Cretaceous-Paleogene extinction event (KT), the Paleocene-Eocene Thermal Maximum (PETM), the Terminal Tethyan Event (TTE) and the uplift of the Isthmus of Panama (IoP) are highlighted in orange

The fossil-calibrated Bayesian molecular phylogeny presented here (Fig. 4) supports an Early Cretaceous origin for the family Atlantidae and implies that the gap in the atlantid fossil record from the Early Cretaceous to the Oligocene is likely due to poor shell preservation. The fossil record of thin aragonitic holoplanktonic gastropod shells is affected by both dissolution and by compaction during diagenesis [35]. The euthecosome pteropods, of similar shell composition, thickness and size to the atlantids are also greatly affected by diagenetic processes, and have a similar gap within their fossil record, from the Late Cretaceous until the Eocene [35], although recently, several potential shelled pteropods from the Paleogene (Danian) and Late Cretaceous have been described [36]. In modern oceans, pteropods are generally much more abundant than atlantids and this difference in abundance may explain why more pteropod fossils have been found. The fossil record of both atlantids and euthecosome pteropods is more complete from the Eocene to the present day [5, 35]. It is very likely that there are Early Cretaceous to Oligocene atlantid fossils still to be found. Despite the prodigious palaeontological research of Janssen (e.g. [15, 25, 37]) and Nützel (e.g. [16, 20]), there are few researchers working in this field.

### Evolutionary history of the family Atlantidae

The fossil-calibrated Bayesian phylogeny (Fig. 4) has a comparable topology to the 3-gene ML tree (Fig. 3), with each genus of the Atlantidae a well-supported monophyletic group. The Bayesian phylogeny also confirms the grouping of smaller ornamented, and larger less ornamented species of *Atlanta* (posterior probability > 99%)*.* The calibration fossil for the oldest member of the superfamily Pterotracheoidea (heteropods) roots the molecular clock analyses within the Early Jurassic (Fig. 4). The order Littorinimorpha, in which Pterotracheoidea belongs, also originated in the Early Jurassic [38]. Therefore, it is probable that little time transpired between the origins of the Littorinimorpha and Pterotracheoidea groups [16]. These gastropods likely adopted a holoplanktonic lifestyle in response to several environmental pressures that made the seafloor an unfavourable habitat. During the Marine Mesozoic Revolution (251.9–66.0 Ma) a re-shuffling of the marine realm took place [17]. Throughout the Jurassic and Cretaceous, gastropods underwent great diversification, with benthic gastropods generally showing a strengthening of their shell that is thought to have been caused by increased predation pressure [17]. Therefore, a move to a holoplanktonic lifestyle for gastropods such as *Coelodiscus minutus* may have been an escape from benthic predators. Anoxic bottom waters are also known to have occurred during the Jurassic [16]. Teichert and Nützel [16] suggest that increasing frequency of dysoxic episodes and hostile benthic conditions likely stimulated planktotrophic gastropod larvae to extend their planktonic phase, eventually evolving into holoplanktonic species. Elsewhere in the marine realm during the Jurassic there was a clear expansion and diversification of marine microplankton with the appearance of the first planktonic foraminifera and coccolithophores [17], suggesting favourable conditions for calcifiers to colonise the upper ocean.

The molecular dating analyses propose a Mid-Cretaceous Albian origin for the family Atlantidae (109–101 Ma, Fig. 4, Table 2). This was a time of widespread changes in the ocean climate system driven by tectonic processes and volcanism. Sea levels rose, and increased atmospheric CO_2_ induced global warming, leading to changes in ocean circulation and ocean stratification [39]. With the continuation of the Marine Mesozoic Revolution, there was a turnover of calcifying marine plankton with high rates of extinction accompanied by a dramatic increase in speciation and plankton diversity [39]. In the Albian, ocean carbonate chemistry was altered, potentially through hydrothermal activity, and began to favour calcium carbonate producing plankton [40]. There was a marked increase in marine productivity and calcification in planktonic foraminifera and calcareous nanoplankton despite the rise in atmospheric CO_2_. This was potentially made possible by nutrient supply from submarine volcanism and a greater flux of nutrients to the ocean from intensified terrestrial weathering [39].

**Table 2:** Overview of calibrated node ages resulting from two independent runs of 10^8^ generations (for nodes with a fossil calibration), and derived node ages either resulting from independent runs that did not include a calibration for that node (for nodes with a fossil calibration), or derived from two independent runs using all calibrations (all nodes without a fossil calibration). A derived age was not possible for the node calibrated by *Atlantidea rotundata* because the tree topology changed when this fossil was removed

Node	Calibration type	Calibration age (Ma)	Calibrated or derived age	Age of crown (95% confidence intervals, Ma)
Earliest heteropod	Fossil: *Coelodiscus minutus*	190.8–170.3	Calibrated	185.39 (189.76–182.86)
			Derived	113.68 (138.62–100.83)
Family Atlantidae	Fossil: *Bellerophina minuta*	113–100.5	Calibrated	103.44 (109.19–100.59)
			Derived	46.71 (75.23–27.10)
Genus *Atlanta*	Fossil: *Atlanta* sp.	28.1–23.03	Calibrated	25.46 (28.46–23.42)
			Derived	35.88 (58.41–16.17)
Clade *Oxygyrus+* *Protatlanta*	Fossil: *Atlantidea rotundata*	15.97–13.82	Calibrated	19.18 (24.22–15.47)
			Derived	N/A
Genus *Protatlanta*	Fossil: *Protatlanta kbiraensis*	15.97–13.82	Calibrated	14.50 (15.32–13.92)
			Derived	8.08 (12.53–3.59)
Genus *Oxygyrus*			Derived	8.11 (13.04–3.66)
*Atlanta brunnea* group			Derived	10.92 (15.95–5.84)
*Atlanta inflata* group			Derived	15.69 (21.41–9.88)
*Atlanta lesueurii* group			Derived	16.48 (21.50–11.62)
*Atlanta peronii* group			Derived	15.32 (20.62–9.80)
*Atlanta gaudichaudi* group			Derived	13.35 (19.34–7.56)
*Atlanta inclinata* group			Derived	4.75 (9.78–1.10)
*Atlanta gibbosa* group			Derived	11.78 (17.64–6.31)
Clade *Atlanta peronii*	Fossil: *Atlanta peronii*	3.6–2.58	Calibrated	3.24 (3.98–2.72)
			Derived	9.38 (15.27–4.03)

Due to the limited atlantid fossil record, it is not possible to determine whether the atlantids diversified during the Late Cretaceous, Paleocene or Eocene, only that the ancestor of *Protatlanta* and *Oxygyrus* likely split from the ancestor of *Atlanta* in the Mid-Cretaceous (Albian, Fig. 4). However, both extant lineages of the family Atlantidae do persist over the Cretaceous-Paleogene (KPg or KT, ~ 66 Ma) extinction event and the Paleocene-Eocene Thermal Maximum (PETM, ~ 56 Ma), both times of intense environmental change and ocean acidification [7, 8]. There are no known Paleocene fossils for the *Oxygyrus + Protatlanta* lineage, the first known fossil being *Atlantidea rotundata* (Gabb, 1873) in the Oligocene. However, the partial replacement of aragonite with conchiolin in the shells of this lineage may have been a response to ocean acidification. This change is particularly evident in *Oxygyrus* where conchiolin covers the aragonite shell entirely, likely protecting it from seawater chemistry in a similar way to the periostracum in pteropods [41]. All major lineages of the aragonite shelled pteropods also lived through these periods [6, 42], experiencing a reduction in surface ocean pH during the PETM on the order of 0.3–0.4 units [43]. It is thought that euthecosome pteropods even diversified during the PETM [6], possibly in response to increased nutrient levels and ocean warming [43].

Our analyses estimate that from ~ 28–23 Ma to the Present day, considerable radiation of the Atlantidae lineages that lead to Recent species occurred, including the origin of all extant genera and species. In particular, there has been rapid diversification in the genus *Atlanta* during the last ~ 25 Ma (Fig. 4). Estimated times for the common ancestor of the three atlantid genera were 25.46 Ma (28.46–23.42 Ma), 14.50 Ma (15.32–13.92 Ma) and 8.11 Ma (13.04–3.66 Ma) for *Atlanta, Protatlanta* and *Oxygyrus,* respectively (Table 2). This dating supports the morphology based hypothesis that a conchiolin shell and conchiolin keel are more derived characters in the family Atlantidae, and were likely not features of the Late Cretaceous *B. minuta,* which shares a similar shell morphology to the calcified juvenile shell of Recent *Oxygyrus*.

Two important vicariance events occurred in the last 25 Ma, including the Terminal Tethyan Event (TTE) of the Mid-Miocene at ~ 18–12 Ma [44] and the uplift of the Isthmus of Panama (IoP) in the Pliocene at ~ 3 Ma [45]. These geographical events had a pronounced effect on the ocean and the evolution of marine organisms. During the period of oceanographic and climate change surrounding the closure of the Miocene Tethys Sea, which separated the Atlantic Ocean from the Indian Ocean, lineages leading to Recent species of atlantid underwent rapid diversification, with the appearance of all of the modern species groups (except the *Atlanta inclinata* Gray, 1850 group Fig. 4, Table 2). Diversification of the euthecosome pteropods also occurred at this time, with many of the extant genera originating in the Mid-Miocene [42]. For the atlantids, this is also the first extinction event detectable within the fossil record, with three species becoming extinct at ~ 13.82 Ma [5]. Due to the incomplete fossil record of atlantids, it is not possible to estimate what proportion of total atlantid diversity became extinct at this time, however, only *Atlantidea rotundata* and an unidentified *Atlanta* sp. are known to have survived this event [5].

Molecular dating analyses indicate that most modern species arose following the TTE in the Tortonian at ~ 12–10 Ma (Fig. 4). Additionally, our results suggest that several atlantid morphospecies also split into multiple putative species within the last 5 Ma, including *Atlanta peronii* A, B, C1 and C2, *Atlanta rosea* A and B Gray 1850, *Atlanta meteori* A and B Richter, 1972, *Atlanta oligogyra* B and C Tesch, 1906 (Fig. 4). This may have been a response to the Pliocene uplift of the IoP at ~ 3 Ma, which closed the link between the Atlantic Ocean and the Pacific Ocean [45]. All of these putative species (apart from *A. peronii* A and B) show clear geographical separation, with one putative species being present in the Atlantic Ocean only, and the other being present in the Pacific and Indian Oceans [11]. A second large-scale and global (known from deposits in the Caribbean Sea, Mediterranean Sea, Philippines and Japan [5]) atlantid extinction event also took place during this time, with 40% of known atlantid species becoming extinct; five species at the Pliocene-Pleistocene boundary (~ 2.58 Ma) and *Atlanta cordiformis* Gabb, 1873 at ~ 5.33 Ma [5]. Therefore, the rapid diversification of the genus *Atlanta* at this time was probably also accompanied with population bottlenecks and (local) extinctions caused by ocean changes related to the IoP. More recently, localised extinctions of *Atlanta turriculata* and *Atlanta plana* in the Atlantic Ocean, and *Protatlanta sculpta* in the Indian Ocean have been detected in the Quaternary fossil record [12–14]. These extinctions have occurred over the last ~ 24 ka and may reflect ocean changes during the period following the Last Glacial Maximum.

### Conclusions **-** implications for a changing ocean

Calcifying plankton are widely accepted to be amongst the most sensitive and first affected by current and predicted future warming and acidification of the world oceans [46]. The results of this study show that the upper ocean inhabiting, aragonite shell bearing atlantid heteropods were able to persist through past climate crises, including the C-Pg (KT) extinction event, and the PETM. The PETM is of particular interest, as it is considered the most analogous geological event to the current Anthropogene climate crisis. However, the current rates of change are unprecedented, even in comparison to the PETM [47] and there is really no analogous climatic event with which to compare the predicted future conditions [7]. Many marine organisms are unlikely to have sufficient time to adapt at the current rate of change.

The present study shows that although global-scale environmental changes over the last 25 Ma have likely caused large scale extinction of atlantids, they have also resulted in periods of exceptional diversification. Past ocean changes, largely resulting from vicarance events, provided opportunities for speciation and have driven evolution within this family. Vermeij [48] hypothesised that when raw materials (e.g. from submarine volcanism) and energy (e.g. CO_2_ induced global warming) become available to organisms at unusually high rates, there are increased opportunities for evolution and diversification. Planktonic organisms have high evolutionary potential and are considered to be well poised for evolutionary responses to global change [49]. Therefore, if atlantids are able to keep up with the rate of anthropogenic ocean changes, they may be able to not only survive, but even diversify in the changing ocean.

## Methods

### Specimen collection

A total of 588 specimens from all 34 known putative atlantid species [11], including all 24 currently described atlantid morphospecies, and 15 specimens of one carinarid species were analysed in this study (591 CO1, 240 28S and 234 18S sequences, Table S1, Fig. S4). For the concatenated gene phylogeny, a subset of 102 specimens was selected to provide good geographical coverage for each known putative species (Fig. S4). Of these specimens, 36 were collected from 18 stations in the Atlantic Ocean during the Atlantic Meridional Transect cruises in 2014 (AMT24, *N* = 28) and 2017 (AMT27, *N* = 8). A total of 24 specimens were collected from nine stations in the Indian Ocean during oceanographic cruises SN105 (*N* = 18) and VANC10MV (*N* = 6). In the Pacific Ocean, 42 specimens were collected from 23 stations during cruises ACE-ASIA (N = 1), DRFT (N = 2), KH1110 (*N* = 16), KM1109 (N = 2), KOK1703 (*N* = 3), S226 (*N* = 4), SO255 (*N* = 13) and WCOA16 (N = 1). In addition, for the concatenated gene phylogeny, two specimens of the partially shelled heteropod genus *Carinaria*, collected during the oceanographic cruise SN105 (N = 2), were used as outgroup taxa.

Collection methods included the use of a variety of plankton nets (e.g. ring, bongo, midwater trawl). These have been previously described for most of the oceanographic cruises listed here [11, 50–54]. Collection techniques for cruises SO255, KOK1703 and AMT27 have not been previously published and we describe them here. Cruise SO255 took place on board the RV *Sonne* to the north east of New Zealand between March and April 2017. Specimens were collected using a ring net with an aperture of 1 m diameter, a mesh size of 350 μm and a maximum sampling time of 30 min. At stations SO255_041 and SO255_057, vertical net hauls were carried out from 200 m water depth to the surface. At all other stations, oblique tows were made in the upper 100 m. Cruise KOK1703 took place offshore of Hawaii, around station ALOHA, on board the RV *Ka’imikai-O-Kanaloa* in March 2017. Sampling was carried out using either a 0.71 m diameter CalBOBL bongo net with a mesh size of 200 μm, or a ring net with a 2 m diameter and mesh size 505 μm. For all stations, oblique tows were conducted in the upper 200 m for a maximum of 44 min. During cruise AMT27, specimens were collected using a 0.71 m diameter bongo net and a 1 m diameter ring net, both with a mesh size of 200 μm. Oblique bongo net tows sampled a range of maximum depths from 233 to 388 m and ring net tows sampled a range of maximum depths from 56 to 99 m.

### DNA extraction and amplification

Prior to DNA extraction, all specimens were imaged using stacking microscopy on a Zeiss Discovery V20 or V12 microscope (images for previously unpublished specimens deposited in the Barcode of Life Data System, BOLD, accession numbers in Table S1). DNA was extracted from whole specimens using two methods. For most specimens, the NucleoMag 96 Tissue kit (Macherey-Nagel) was used on a Thermo Scientific KingFisher Flex magnetic particle processor, with a final elution volume of 75 μl. For smaller batches of specimens, DNA was extracted using the DNeasy Blood and Tissue spin-column protocol with a final elution volume of 100 μL. Both methods were successful. An archive of DNA extracts, collection information and images for all specimens is stored at Naturalis Biodiversity Center, Leiden under the accession numbers in Supplementary Table 1.

Three commonly used gene fragments were amplified. A ~ 570 bp fragment of the mitochondrial cytochrome *c* oxidase subunit 1 gene (CO1) was amplified using primers jgLCO1490 (5′–TITCIACIAAYCAYAARGAYATTGG–3′) and jgHCO2198 (5′–TAIACYTCIGGRTGICCRAARAAYCA–3′) [55]. A ~ 1000 bp fragment of nuclear 28S rRNA was amplified using primers C1-F (5′-ACCCGCTGAATTTAAGCAT-3′) [56] and D3-R (5′-GACGATCGATTTGCACGTCA-3′) [57]. A ~ 970 bp fragment of nuclear 18S rRNA was amplified using primers 18S-KP-F (5′-TGGAGGGCAAGTCTGGTG-3′) [42] and 1800R (5′-GATCCTTCCGCAGGTTCACCTACG-3′) [57]. Primers were tailed with M13F and M13R for sequencing [58].

All PCR reactions contained 17.30 μl ultra pure water (mQ), 2.50 μl 10x Qiagen PCR buffer, 0.50 μl 25 mM MgCl2, 1.00 μl 100 mM BSA, 1.00 μl 10 mM of each primer, 0.50 μl 2.5 mM dNTPs and 0.25 μl 5 U Qiagen Taq, with 1.00 μl of template DNA, which was diluted up to 100 times for some samples. The same PCR steps were performed for all three genes using an initial denaturation step of 180 s at 96 °C, followed by 40 cycles of 15 s at 96 °C, 30 s at 50 °C and 40 s at 72 °C, and finishing with a final extension of 300 s at 72 °C. Sequencing of forward and reverse strands was carried out by either Macrogen Europe (Amsterdam), or Baseclear (Leiden).

### Phylogenetic analyses

For individual gene phylogenies, a total of 237 new 28S sequences, 230 new 18S sequences, and 99 new and 477 previously published [11, 59, 60] CO1 sequences of atlantids were included (Table S1). The genus *Carinaria* from the closely related heteropod family Carinariidae was used as an outgroup with 14 CO1, three 28S and four 18S sequences included from 15 specimens collected in the Indian Ocean. Here, we consider a ‘putative species’ to be a well-supported monophyletic group (bootstrap support > 80%) in our CO1 phylogenetic tree, with interspecific genetic distances of > 6.95% (Jukes-Cantor). We verified our selection of putative species using Automatic Barcode Gap Discovery (ABGD) performed on the entire CO1 dataset using Jukes-Cantor genetic distances with default settings [61]. Similar to previous ABGD analysis of atlantid CO1 sequences [11], *Atlanta inclinata* and *Atlanta tokiokai* van der Spoel & Troost, 1972 were grouped together due to high intraspecific genetic diversity. However, here we consider them to be two separate species because of clear morphological differences. All other previously identified putative species [11] were recognised by the ABGD analysis. Two additional putative species were also confirmed, one within *A. peronii* C, and one within *O. inflatus* A, bringing the total number of putative species to 36.

For the concatenated gene (3-gene) phylogeny, at least one specimen from each atlantid putative species was included from each ocean basin in which that putative species resides. As far as possible, all three genes were combined from a single specimen. In two cases, for *Atlanta plana* and *Atlanta selvagensis*, it was necessary to combine genes from different specimens at the same station (*A. plana* SN105 station 19, *A. selvagensis* AMT24 station 16), in order to obtain complete taxon sampling for all genes. Where records have been combined, specimens were confirmed to be conspecific using CO1, which was available for all specimens. All three markers were obtained for each putative species in each region, except for *Atlanta gaudichaudi*, which was only successful for CO1 and 18S, and *Atlanta californiensis* for which only CO1 was sequenced. A total of 102 CO1, 100 28S and 101 18S sequences are included in the concatenated gene phylogeny.

New sequences were verified and edited using Geneious R8 and all sequences were aligned using MEGA 7 [62]. All gaps in the alignments of 28S and 18S were trimmed resulting in final alignments of 851 bp for 28S and 964 bp for 18S. The alignment of CO1 was checked for stop codons and then all 657 sites were included in the analyses. The concatenated gene alignment was a total of 2472 bp.

Single gene and 3-gene phylogenetic relationships were resolved using maximum likelihood analyses in RaxmlGUI 1.5b2 [63]. Using jModelTest, the most appropriate evolutionary model was determined to be the General Time Reversible (GTR) model with a proportion of invariable sites (+I) and gamma distributed rate variation among sites (+G) independently for each gene as well as for the 3-gene alignment. For the single gene phylogenies, a maximum likelihood search was performed with thorough bootstrapping analysis of 1000 replicates applied for CO1, and 1500 replicates applied for 28S and 18S. The same analysis was carried out for the concatenated gene analysis, but with the three genes partitioned and 3000 replicates applied.

### Fossil-calibrated phylogeny

A subset of 34 atlantid and one *Carinaria* concatenated gene sequences were included in the fossil-calibrated phylogeny, including a single representative of each putative species identified in previous CO1 phylogenies [11] (35 CO1, 33 28S, 34 18S). Fossil-calibrated analysis was carried out using BEAST 2.5.0 [64]. A GTR site model and relaxed log-normal molecular clock were applied in BEAUti 2.5.0. A Yule model was calibrated using log-normal distributions of several fossil dates (summarised in Table 3). The crown node of the superfamily Pterotracheoidea was calibrated using the earliest potential heteropod, *Coelodiscus minutus* [16] from the Pliensbachian of the Early Jurassic (190.8–182.7 Ma). For the remaining calibration, the oldest known fossils for the family Atlantidae (*Bellerophina minuta* [23]) and the genera *Atlanta* (*Atlanta* sp. [25]) and *Protatlanta* (*Protatlanta kbiraensis* [25]) were used as crown calibrations (Table 3). The Eocene genus *Eoatlanta* and the species *Eoatlanta spiruloides* were not included in the calibrations because *E. spiruloides* is now thought to be a benthic gastropod in the superfamily Vanikoroidea [65, 66]. Based on careful consideration of morphology, the extinct species *Atlantidea rotundata* [26] was used to calibrate the node *Oxygyrus + Protatlanta*. This species exhibits morphological characters in common with both genera, having larval shell ornamentation similar to *Oxygyrus*, but an adult shell similar to *Protatlanta,* and a smooth periphery suggesting a conchiolin keel (common to *Oxygyrus* and *Protatlanta*)*.* Running the Bayesian analysis without the *Atlantidea rotundata* calibration changed the topology of the tree, grouping *Atlanta* with *Oxygyrus.* This placement is not in agreement with the topology of the concatenated ML phylogeny, which shows that relationships between the three genera are not well resolved with the current molecular dataset. Finally, the morphospecies *Atlanta peronii,* which is made up of four putative species, was used to calibrate more recent speciation [15]. The fossil genus *Freboldia* (163.5–157.3 Ma) was not included in the calibrations because, unlike *B. minuta,* this species does not show morphological similarity to the Atlantidae and we are unconfident in its current placement [18]. Two independent MCMC chains were run with 10^8^ generations each and checked for convergence with a burnin of 10% in Tracer 1.6.0 [67] before being combined using LogCombiner 2.5.0 [64]. Trees and log-likelihood values were sampled at every 10^4^ generations. Maximum clade credibility trees were selected using TreeAnnotator 2.5.0 [64]. Calibration points were checked by running multiple analyses and leaving out one calibration fossil each time (Table 2).

**Table 3:** Settings for the molecular clock calibration using a Yule model

Calibrated node	Calibration type	Age	Oldest age (Ma)	Youngest age (Ma)	M	S	Offset	Prior dist.	Reference
Superfamily Pterotracheoidea	Fossil: *Coelodiscus minutus*	Pliensbachian	190.8	170.3	3.00	0.80	182.7	Log-normal	[8]
Family Atlantidae	Fossil: *Bellerophina minuta*	Albian	113	100.5	4.00	1.00	100.5	Log-normal	[14]
Genus *Atlanta*	Fossil: *Atlanta* sp.	Chattian	28.1	23.03	2.40	0.55	23.03	Log-normal	[16]
Clade *Oxygyrus+* *Protatlanta*	Fossil: *Atlantidea rotundata*	Langhian	15.97	13.82	0.90	0.65	13.82	Log-normal	[17]
Genus *Protatlanta*	Fossil: *Protatlanta kbiraensis*	Langhian	15.97	13.82	0.90	0.65	13.82	Log-normal	[16]
Clade *Atlanta peronii*	Fossil: *Atlanta peronii*	Piacenzian	3.6	2.58	0.51	0.50	2.58	Log-normal	[11]

## Supplementary information

**Additional file 1: Supplementary Table 1.** Specimens included in this study.

**Additional file 2: Supplementary Figure 1.** Maximum likelihood phylogeny of the family Atlantidae based on cytochrome *c* oxidase subunit 1 mitochondrial DNA (CO1). Black squares represent bootstrap support > 80%. Species groups based on morphology are highlighted with coloured boxes (See Table 1).

**Additional file 3: Supplementary Figure 2.** Maximum likelihood phylogeny of the family Atlantidae based on the nuclear gene 28S. Poorly supported branches (< 60%) have been collapsed to simplify the phylogeny. Black squares represent bootstrap support > 80%. Species groups based on morphology are highlighted with coloured boxes (See Table 1).

**Additional file 4: Supplementary Figure 3.** Maximum likelihood phylogeny of the family Atlantidae based on the nuclear gene 18S. Poorly supported branches (< 60%) have been collapsed to simplify the phylogeny. Black squares represent bootstrap support > 80%. Species groups based on morphology are highlighted with coloured boxes (See Table 1).

**Additional file 5 : Supplementary Figure 4.** The distribution of all specimens used in this study demonstrates the global coverage of the dataset. Filled circles represent specimens used for the concatenated gene phylogeny. Data were visualised using the software QGIS v2.8 (https://www.qgis.org/en/site/).

## Data Availability

The molecular dataset supporting the results of this article, including specimen images and collection information, is available from the Barcode of Life Data System (BOLDSYSTEMS) repository [68] doi:10.5883/DS-ATLANTID. Individual specimen accession numbers can be found in Supplementary Table 1. Single gene and concatenated alignments are available from Figshare [69] doi:10.6084/m9.figshare.12420365. Voucher DNA extracts are held at the Naturalis Biodiversity Center (bioportal.naturalis.nl) under museum accession numbers presented in Supplementary Table 1.

## Abbreviations

ABGD: Automatic Barcode Gap Discovery
AMT24 and AMT27: Atlantic Meridional Transect cruises for the years 2014 and 2017
BOLD or BOLDSYSTEMS: Barcode of Life data system
CO1: cytochrome *c* oxidase subunit 1
CO_2_: carbon dioxide
DNA: deoxyribonucleic acid
GTR: General Time Reversible
IoP: the uplift of the Isthmus of Panama
KPg or KT: Cretaceous-Paleogene extinction event
PETM: Paleocene Eocene Thermal Maximum
rRNA: ribosomal ribonucleic acid
TTE: Terminal Tethyan Event of the Mid-Miocene at ~ 18–12 Ma

## References

[1] Karakas F, D’Oliveira D, Maas AE, Murphy DW (2018). Using a shell as a wing: pairing of dissimilar appendages in atlantiid heteropod swimming. J Exp Biol.

[2] Lalli CM, Gilmer RW (1989). Pelagic snails: the biology of holoplanktonic mollusks.

[3] Newman LJ (1990). The taxonomy, distribution and biology of *Atlanta gaudichaudi* Souleyet, 1852 (Gastropoda, Heteropoda) from the great barrier reef, Australia. Am Malacological Bull.

[4] Thiriot-Quiévreux C (1973). Heteropoda. Oceanogr Mar Biol Annu Rev.

[5] Wall-Palmer D, Smart CW, Kirby R, Hart MB, Peijnenburg KTCA, Janssen AW (2016). A review of the ecology, palaeontology and distribution of atlantid heteropods (Caenogastropoda: Pterotracheoidea: Atlantidae). J Molluscan Stud.

[6] Peijnenburg KTCA, Janssen AW, Wall-Palmer D, Goetze E, Maas A, Todd JA, et al. The origin and diversification of pteropods precede past perturbations in the Earth’s carbon cycle. Proc Natl Acad Sci. In press. 10.1073/pnas.1920918117 PMC7568333 32973093

[7] Zeebe RE, Ridgwell A, Zachos JC (2016). Anthropogenic carbon release rate unprecedented during the past 66 million years. Nat Geosci.

[8] Henehan MJ, Ridgwell A, Thomas E, Zhang S, Alegret L, Schmidt DN (2019). Rapid Ocean acidification and protracted earth system recovery followed the end-cretaceous Chicxulub impact. Proc Natl Acad Sci.

[9] Manno C, Bednaršek N, Tarling GA, Peck VL, Comeau S, Adhikari D (2017). Shelled pteropods in peril: assessing vulnerability in a high CO 2 ocean. Earth Sci Rev.

[10] Wall-Palmer D, Mekkes L, Ramos-Silva P, Dämmer L, Goetze E, Bakker K, et al. Current and future ocean chemistry negatively impacts calcification in predatory planktonic snails. bioRxiv. 2020. 10.1101/2020.08.04.236166.

[11] Wall-Palmer D, Burridge AK, Goetze E, Stokvis FR, Janssen AW, Mekkes L (2018). Biogeography and genetic diversity of the atlantid heteropods. Prog Oceanogr.

[12] Wall-Palmer D, Smart CW, Hart MB (2015). Data report: the Late Quaternary fossil record of holoplanktonic gastropods at IODP sites U1395 and U1394. Proceedings of the Integrated Ocean Drilling Program.

[13] Janssen AW (2004). Holoplanktonic molluscan assemblages (Gastropoda, Heteropoa, Thecosomata) from the Pliocene of Estepona (Spain, Málaga). Palaeontos..

[14] Janssen AW (2012). Early Pliocene heteropods and pteropods (Mollusca, Gastropoda) from Le Puget-Sur-Argens (Var), France. Cainozoic Res.

[15] Janssen AW. Holoplanktonic Mollusca Gastropoda: Pterotracheoidea, Janthinoidea, Thecosomata and Gymnosomata) from the Pliocene of Pangasinan (Luzon, Philippines). Scr Geol 2007;135:29–177.

[16] Teichert S, Nützel A. Early Jurassic anoxia triggered the evolution of the oldest holoplanktonic gastropod Coelodiscus minutus by means of heterochrony. Acta Palaeontol Pol. 2015. 10.4202/app.00145.2014.

[17] Vermeij GJ (1977). The Mesozoic marine revolution: evidence from snails, Predators and Grazers. Paleobiology.

[18] Nützel A, Schneider S, Hülse P, Kelly SRA, Tilley L, Veit R (2016). A new early Jurassic gastropod from Ellesmere Island, Canadian Arctic – an ancient example of holoplanktonic gastropods. Bull Geosci.

[19] Bandel K, Hemleben C (1987). Jurassic heteropods and their modern counterparts (planktonic Gastropoda, Mollusca). Neues Jahrb Geol Palaontol Abh.

[20] Nützel A, Gründel J. Early Jurassic (Pliensbachian) gastropods from Franconia, Southern Germany. Palaeontographica Abteilung A. 2015;305:1–89.

[21] Schulbert C, Nützel A (2013). Gastropods from the early/middle Jurassic transition of Franconia (southern Germany). Bull Geosci.

[22] Tracey S (2010). Gastropods. Fossils of the Gault clay.

[23] Sowerby J. The mineral conchology of Great Britain, Vol. 1, part 10. London: Sowerby; 1814.

[24] Destombes P (1984). Recherches sur la mésofaune de l’Albien inférieur de Bully-Saint-Martin l’Horbier (Pays de Bray). Bulletin Trimestriel de la Société Géologique de Normandie et des Amis du Musée du Havre.

[25] Janssen AW (2012). Systematics and biostratigraphy of holoplanktonic mollusca from the Oligo-Miocene of the Maltese archipelago. Museo Regionale di Scienze Naturali Torino, Bullettino.

[26] Pilsbury HA. Revision of W.M. Gabb’s Tertiary Mollusca of Santa Domingo. Proceedings of the Academy of Natural Sciences of Philadelphia. 1922;73:305–435.

[27] Richter G (1961). Die radula der Atlantiden (Heteropoda, prosobranchia) und ihre Bedeutung für die Systematik und Evolution der Familie. Z Morphol Okol Tiere.

[28] Richter G (1974). Die Heteropoden der Meteor Expedition in den Indischen Ozean 1964/65. Meteor Forschungsergebnisse.

[29] Richter G (1968). Heteropoden und Heteropodenlarven im Oberflächenplankton des Golfs von Neapel. Pubblicazioni della Stazione Zoologica di Napoli.

[30] Richter G. Zur Stammesgeschichte pelagischer Gastropoden. Natur und Museum. 1973;103:265–75.

[31] Seapy RR. Atlantidae. Tree of Life web project. 2011. http://tolweb.org/Atlantidae. Accessed 10 July 2019.

[32] Wall-Palmer D, Hegmann M, Goetze E, Peijnenburg KTCA (2019). Resolving species boundaries in the *Atlanta brunnea* species group (Gastropoda, Pterotracheoidea). ZooKeys..

[33] Seapy RR. Atlantidae Rang, 1829. Version 01 April 2010 (under construction). http://tolweb.org/Atlantidae/28732/2010.04.01 in The Tree of Life Web Project, http://tolweb.org/. 2010.

[34] Thiriot-Quiévreux C, Seapy RR (1997). Chromosome studies of three families of pelagic heteropod molluscs (Atlantidae, Carinariidae, and Pterotracheidae) from Hawaiian waters. Can J Zool.

[35] Janssen AW, Peijnenburg KTCA (2017). An overview of the fossil record of Pteropoda (Mollusca, Gastropoda, Heterobranchia). Cainozoic Res.

[36] Garvie CL, Goedert JL, Janssen AW (2020). Paleogene and late cretaceous Pteropoda (Mollusca, Gastropoda, Heterobranchia) from North America. Zootaxa..

[37] Janssen AW (2012). Late Quaternary to recent holoplanktonic Mollusca (Gastropoda) from bottom samples of the eastern Mediterranean Sea: systematics, morphology. Bollettino Malacologico.

[38] Zapata F, Wilson NG, Howison M, Andrade SCS, Jorger KM, Schrodl M (2014). Phylogenomic analyses of deep gastropod relationships reject Orthogastropoda. Proc R Soc B Biol Sci.

[39] Leckie RM, Bralower TJ, Cashman R (2002). Oceanic anoxic events and plankton evolution: Biotic response to tectonic forcing during the mid-Cretaceous: oceanic anoxic events and plankton evolution. Paleoceanography.

[40] Stanley SM, Hardie LA (1998). Secular oscillations in the carbonate mineralogy of reefbuilding and sediment-producing organisms driven by tectonically forced shifts in seawater chemistry. Palaeogeogr Palaeoclimatol Palaeoecol.

[41] Peck VL, Tarling GA, Manno C, Harper EM, Tynan E (2016). Outer organic layer and internal repair mechanism protects pteropod *Limacina helicina* from ocean acidification. Deep-Sea Res II Top Stud Oceanogr.

[42] Burridge AK, Hörnlein C, Janssen AW, Hughes M, Bush SL, Marlétaz F (2017). Time-calibrated molecular phylogeny of pteropods. PLoS One.

[43] Janssen AW, Sessa JA, Thomas E (2016). Pteropoda (Mollusca, Gastropoda, Thecosomata) from the Paleocene-Eocene Thermal Maximum (United States Atlantic Coastal Plain). Palaeontologia Electron.

[44] Cowman PF, Bellwood DR (2013). Vicariance across major marine biogeographic barriers: temporal concordance and the relative intensity of hard versus soft barriers. Proc R Soc B Biol Sci.

[45] O’Dea A, Lessios HA, Coates AG, Eytan RI, Restrepo-Moreno SA, Cione AL (2016). Formation of the isthmus of Panama. Sci Adv.

[46] Guinotte JM, Fabry VJ (2008). Ocean acidification and its potential effects on marine ecosystems. Ann N Y Acad Sci.

[47] Zeebe RE, Zachos JC (2013). Long-term legacy of massive carbon input to the earth system: Anthropocene versus Eocene. Philosophical transactions of the Royal Society a: mathematical, physical and. Eng Sci.

[48] Vermeij GJ (1995). Economics, volcanoes, and Phanerozoic revolutions. Paleobiology..

[49] Peijnenburg KTCA, Goetze E (2013). High evolutionary potential of marine zooplankton. Ecol Evol.

[50] Burridge AK, Goetze E, Wall-Palmer D, Le Double SL, Huisman J, Peijnenburg KTCA (2017). Diversity and abundance of pteropods and heteropods along a latitudinal gradient across the Atlantic Ocean. Prog Oceanogr.

[51] Goetze E (2005). Global population genetic structure and biogeography of the oceanic copepods *Eucalanus hyalinus* and *E. spinifer*. Evolution..

[52] Goetze E (2003). Cryptic speciation on the high seas; global phylogenetics of the copepod family Eucalanidae. Proc R Soc Lond Ser B Biol Sci.

[53] Halbert KMK, Goetze E, Carlon DB (2013). High Cryptic Diversity across the Global Range of the Migratory Planktonic Copepods *Pleuromamma piseki* and *P gracilis*. Plos One.

[54] Hirai J, Tsuda A, Goetze E (2015). Extensive genetic diversity and endemism across the global range of the oceanic copepod *Pleuromamma abdominalis*. Prog Oceanogr.

[55] Geller J, Meyer C, Parker M, Hawk H (2013). Redesign of PCR primers for mitochondrial cytochrome *c* oxidase subunit I for marine invertebrates and application in all-taxa biotic surveys. Mol Ecol Resour.

[56] Dayrat B, Tillier A, Lecointre G, Tillier S (2001). New clades of Euthyneuran Gastropods (Mollusca) from 28S rRNA sequences. Mol Phylogenet Evol.

[57] Vonnemann V, Schrödl M, Klussmann-Kolb A, Wägele H (2005). Reconstruction of the phylogeny of the Opisthobranchia (Mollusca: Gastropoda) by means of 18S and 28S rRNA gene sequences. J Molluscan Stud.

[58] Messing J (1983). New M13 vectors for cloning. Methods Enzymol.

[59] Wall-Palmer D, Burridge AK, Peijnenburg KTCA (2016). *Atlanta ariejansseni*, a new species of shelled heteropod from the southern subtropical convergence zone (Gastropoda, Pterotracheoidea). ZooKeys..

[60] Wall-Palmer D, Burridge AK, Peijnenburg KTCA, Janssen AW, Kirby R, Hart MB (2016). Evidence for the validity of *Protatlanta sculpta* (Gastropoda: Pterotracheoidea). Contrib Zool.

[61] Puillandre N, Lambert A, Brouillet S, Achaz G (2012). ABGD, Automatic Barcode gap Discovery for primary species delimitation: ABGD, AUTOMATIC BARCODE GAP DISCOVERY. Mol Ecol.

[62] Kumar S, Stecher G, Tamura K (2016). MEGA7: molecular evolutionary genetics analysis version 7.0 for bigger datasets. Mol Biol Evol.

[63] Silvestro D, Michalak I (2012). raxmlGUI: a graphical front-end for RAxML. Organ Divers Evol.

[64] Bouckaert R, Heled J, Kühnert D, Vaughan T, Wu C-H, Xie D (2014). BEAST 2: a software platform for Bayesian evolutionary analysis. PLoS Comput Biol.

[65] Lozouet P (2012). Position systematique de quelques gasteropodes de l’Eocene a dernier tour disjoint (Mollusca, Gastropoda, Caenogastropoda): *Delphinula conica, Omalaxis, Eoatlanta*. Cossmanniana.

[66] Schnetler KI (2013). Eoatlanta ravni nov. sp. (Mollusca: Gastropoda, ?Hipponicidae) from the Danian (early Paleocene) of Faxe, Denmark. Cainozoic Res.

[67] Rambaut A, Suchard M, Xie D, Drummond A. Tracer v1. 6. 2014. http://beast.bio.ed.ac.uk. Accessed 16 July 2019.

[68] Wall-Palmer D, Janssen AW, Goetze E, Choo LQ, Mekkes L, Peijnenburg KTCA. Molecular data from: fossil-calibrated molecular phylogeny of atlantid heteropods (Gastropoda, Pterotracheoidea). Barcode Life Data Syst Repository. 2020. 10.5883/DS-ATLANTID. 10.1186/s12862-020-01682-9 PMC7507655 32957910

[69] Wall-Palmer D, Janssen AW, Goetze E, Choo LQ, Mekkes L, Peijnenburg KTCA. Gene alignments from: fossil-calibrated molecular phylogeny of atlantid heteropods (Gastropoda, Pterotracheoidea). Figshare Digital Repository. 2020. 10.6084/m9.figshare.12420365. 10.1186/s12862-020-01682-9 PMC7507655 32957910

