# Supplementary material for: Fossil-calibrated molecular phylogeny of atlantid heteropods (Gastropoda, Pterotracheoidea)
Source: BMC Evol Biol. 2020 Sep 21;20:124. doi: 10.1186/s12862-020-01682-9 (PMC7507655; doi:10.1186/s12862-020-01682-9)
Supplement: Supplementary file 1 — Additional file 1: Supplementary Table 1. Specimens included in this study. [file 12862_2020_1682_MOESM1_ESM.docx]

**Supplementary material**

Supplementary Table 1. Specimens included in this study.

| Species | Ocean | Cruise | Station | Latitude | Longitude | no. CO1 sequences | no. 28S sequences | no. 18S sequences | Combined gene phylogeny | Time calibrated phylogeny | BOLD process ID or GenBank (GB) accession number | Reference | RMNH |
| --- | --- | --- | --- | --- | --- | --- | --- | --- | --- | --- | --- | --- | --- |
| *Atlanta ariejansseni* | Atlantic | AMT24 | 26 | -37.89 | -28.74 | 2 |  |  |  |  | KX343177 - KX343178 (GB) | Wall-Palmer et al. 2016 | RMNH.MOL.341284, RMNH.MOL.341286 |
| *Atlanta ariejansseni* | Atlantic | AMT24 | 27 | -40.12 | -30.91 | 5 | 1 | 1 | x | x | KX343179 - KX343183 (GB), ATCP364-19 | Wall-Palmer et al. 2016 + New | RMNH.MOL.341282, RMNH.MOL.341293, RMNH.MOL.341289, RMNH.MOL.341296, RMNH.MOL.341295 |
| *Atlanta ariejansseni* | Atlantic | AMT24 | 28 | -41.48 | -33.86 | 1 |  |  |  |  | KX343184 (GB) | Wall-Palmer et al. 2016 | RMNH.MOL.341287 |
| *Atlanta ariejansseni* | Atlantic | AMT24 | 29 | -43.02 | -37.14 | 7 | 2 | 2 |  |  | KX343185 - KX343191 (GB), ATCP365-19, ATCP366-19 | Wall-Palmer et al. 2016 + New | RMNH.MOL.341288, RMNH.MOL.341291, RMNH.MOL.341294, RMNH.MOL.341297, RMNH.MOL.341298, RMNH.MOL.341285, RMNH.MOL.341283 |
| *Atlanta ariejansseni* | Pacific | DRFT | 14 | -38.32 | -161.14 | 2 | 1 | 1 | x |  | KX343192 - KX343193 (GB), ATCP367-19 | Wall-Palmer et al. 2016 + New | RMNH.MOL.341290, RMNH.MOL.341292 |
| *Atlanta brunnea* | Atlantic | AMT24 | 5 | 34.75 | -26.62 | 1 | 1 | 1 | x | x | AGD001-17 | Wall-Palmer et al. 2018 + New | RMNH.MOL.341299 |
| *Atlanta brunnea* | Atlantic | AMT27 | 9 | -6.87 | -25.04 | 1 | 1 |  |  |  | ATCP003-19 | New | RMNH.MOL.341308 |
| *Atlanta brunnea* | Atlantic | AMT27 | 37 | -6.87 | -25.04 | 1 |  |  |  |  | ATCP004-19 | New | RMNH.MOL.341309 |
| *Atlanta brunnea* | Atlantic | AMT27 | 41 | -12.63 | -25.05 | 1 |  | 1 |  |  | ATCP006-19 | New | RMNH.MOL.341311 |
| *Atlanta brunnea* | Atlantic | AMT27 | 47 | -24.00 | -25.02 | 1 |  |  |  |  | ATCP007-19 | New | RMNH.MOL.341312 |
| *Atlanta brunnea* | Pacific | KH1110 | 5 | -23.00 | 180.01 | 1 | 1 |  |  |  | AGD002-17 | Wall-Palmer et al. 2018 + New | RMNH.MOL.341313 |
| *Atlanta brunnea* | Pacific | KOK1703 | 7 | 23.62 | -157.61 | 2 | 2 |  |  |  | ATCP001-19, ATCP002-19 | New | RMNH.MOL.341306, RMNH.MOL.341307 |
| *Atlanta brunnea* | Indian | SN105 | 4 | 8.02 | 67.08 | 2 |  | 1 |  |  | AGD008-17 - AGD009-17 | Wall-Palmer et al. 2018 + New | RMNH.MOL.341304, RMNH.MOL.341305 |
| *Atlanta brunnea* | Indian | SN105 | 8 | 4.38 | 67.00 | 4 | 1 | 1 | x |  | AGD010-17 - AGD013-17 | Wall-Palmer et al. 2018 + New | RMNH.MOL.341300, RMNH.MOL.341301, RMNH.MOL.341302, RMNH.MOL.341303 |
| *Atlanta brunnea* | Pacific | SO255 | 100 | -28.52 | 179.59 | 1 | 1 |  |  |  | ATCP005-19 | New | RMNH.MOL.341310 |
| *Atlanta brunnea* | Pacific | ACAS | 2 | 28.21 | -162.14 |  |  | 1 |  |  | ATCP187-19 | New | RMNH.MOL.341318 |
| *Atlanta vanderspoeli* | Pacific | KH1110 | 15 | -23.00 | -119.27 | 3 | 1 | 1 | x | x | AGD003-17 - AGD005-17 | Wall-Palmer et al. 2018 + New | RMNH.MOL.341320, RMNH.MOL.341321, RMNH.MOL.341322 |
| *Atlanta vanderspoeli* | Pacific | KH1110 | 21 | -23.00 | -100.00 | 3 | 2 | 2 |  |  | AGD006-17 - AGD007-17, ATCP013-19 | Wall-Palmer et al. 2018 + New | RMNH.MOL.341323, RMNH.MOL.341324, RMNH.MOL.341325 |
| *Atlanta vanderspoeli* | Pacific | SO255 | 57 | -29.95 | -178.73 | 1 |  |  |  |  | ATCP012-19 | New | RMNH.MOL.341319 |
| *Atlanta californiensis* | Pacific | WCOA16 | 24 | 31.62 | -116.91 | 1 |  |  |  |  | AGD014-17 | Wall-Palmer et al. 2018 | RMNH.MOL.341336 |
| *Atlanta californiensis* | Pacific | WCOA16 | 30 | 33.16 | -118.42 | 1 |  |  |  |  | AGD015-17 | Wall-Palmer et al. 2018 | RMNH.MOL.341334 |
| *Atlanta californiensis* | Pacific | WCOA16 | 31 | 32.77 | -119.23 | 1 |  |  | x | x | AGD016-17 | Wall-Palmer et al. 2018 | RMNH.MOL.341331 |
| *Atlanta echinogyra* | Pacific | KH1110 | 15 | -23.00 | -119.27 | 1 | 1 | 1 | x |  | AGD017-17 | Wall-Palmer et al. 2018 + New | RMNH.MOL.341350 |
| *Atlanta echinogyra* | Pacific | KH1110 | 21 | -23.00 | -100.00 | 3 | 3 | 3 |  |  | AGD018-17 - AGD020-17 | Wall-Palmer et al. 2018 + New | RMNH.MOL.341351, RMNH.MOL.341352, RMNH.MOL.341353 |
| *Atlanta echinogyra* | Pacific | S226 | 10 | 13.08 | -159.34 | 1 | 1 | 1 |  |  | AGD021-17 | Wall-Palmer et al. 2018 + New | RMNH.MOL.341354 |
| *Atlanta echinogyra* | Indian | SN105 | 4 | 8.02 | 67.08 | 4 |  |  |  |  | AGD022-17 - AGD025-17 | Wall-Palmer et al. 2018 | RMNH.MOL.341343, RMNH.MOL.341344, RMNH.MOL.341345, RMNH.MOL.341341 |
| *Atlanta echinogyra* | Indian | SN105 | 8 | 4.38 | 67.00 | 2 | 1 | 1 |  |  | AGD026-17 - AGD027-17 | Wall-Palmer et al. 2018 + New | RMNH.MOL.341340, RMNH.MOL.341342 |
| *Atlanta echinogyra* | Indian | SN105 | 19 | -2.95 | 66.99 | 3 | 1 | 1 | x | x | AGD028-17 - AGD030-17 | Wall-Palmer et al. 2018 + New | RMNH.MOL.341337, RMNH.MOL.341338, RMNH.MOL.341339 |
| *Atlanta echinogyra* | Pacific | SO255 | 57 | -29.95 | -178.73 | 1 | 1 | 1 |  |  | ATCP022-19 | New | RMNH.MOL.341346 |
| *Atlanta echinogyra* | Pacific | SO255 | 73 | -28.13 | 179.02 | 1 | 1 | 1 | x |  | ATCP023-19 | New | RMNH.MOL.341347 |
| *Atlanta echinogyra* | Pacific | SO255 | 80 | -29.10 | -179.72 | 1 | 1 |  |  |  | ATCP024-19 | New | RMNH.MOL.341348 |
| *Atlanta echinogyra* | Pacific | SO255 | 143 | -32.87 | -179.78 | 1 |  |  |  |  | ATCP025-19 | New | RMNH.MOL.341349 |
| *Atlanta fragilis* | Atlantic | AMT24 | 9 | 20.45 | -29.27 | 1 | 1 | 1 | x |  | ATCP026-19 | New | RMNH.MOL.341360 |
| *Atlanta fragilis* | Atlantic | AMT24 | 13 | 7.29 | -26.49 | 2 | 1 | 1 | x | x | AGD032-17 - AGD033-17 | Wall-Palmer et al. 2018 + New | RMNH.MOL.341374, RMNH.MOL.341378 |
| *Atlanta fragilis* | Atlantic | AMT24 | 19 | -14.66 | -25.07 | 1 |  |  |  |  | AGD034-17 | Wall-Palmer et al. 2018 | RMNH.MOL.341358 |
| *Atlanta fragilis* | Atlantic | AMT24 | 20 | -18.32 | -25.09 | 1 |  |  |  |  | AGD035-17 | Wall-Palmer et al. 2018 | RMNH.MOL.341359 |
| *Atlanta fragilis* | Atlantic | AMT24 | 21 | -20.86 | -25.08 | 1 |  |  |  |  | AGD036-17 (1) | Wall-Palmer et al. 2018 | RMNH.MOL.341373 |
| *Atlanta fragilis* | Atlantic | AMT24 | 22 | -24.46 | -25.04 | 4 | 2 | 2 |  |  | AGD037-17 - AGD040-17 | Wall-Palmer et al. 2018 + New | RMNH.MOL.341377, RMNH.MOL.341355, RMNH.MOL.341356, RMNH.MOL.341357 |
| *Atlanta fragilis* | Atlantic | AMT24 | 27 | -40.12 | -30.91 | 4 | 1 | 1 | x |  | AGD041-17 - AGD044-17 | Wall-Palmer et al. 2018 + New | RMNH.MOL.341375, RMNH.MOL.341376, RMNH.MOL.341379, RMNH.MOL.341372 |
| *Atlanta fragilis* | Indian | VANC | 9 | -31.83 | 52.61 | 1 |  |  |  |  | AGD051-17 | Wall-Palmer et al. 2018 | RMNH.MOL.341363 |
| *Atlanta fragilis* | Indian | VANC | 16 | -19.75 | 78.01 | 1 |  |  |  |  | AGD052-17 | Wall-Palmer et al. 2018 | RMNH.MOL.341364 |
| *Atlanta fragilis* | Indian | VANC | 17 | -18.43 | 80.92 | 1 |  | 1 |  |  | AGD053-17 | Wall-Palmer et al. 2018 + New | RMNH.MOL.341368 |
| *Atlanta fragilis* | Pacific | KH1110 | 15 | -23.00 | -119.27 | 1 |  |  |  |  | AGD046-17 | Wall-Palmer et al. 2018 | RMNH.MOL.341371 |
| *Atlanta fragilis* | Pacific | KH1110 | 18 | -30.00 | -107.00 | 3 |  | 1 |  |  | AGD047-17 - AGD049-17 | Wall-Palmer et al. 2018 + New | RMNH.MOL.341366, RMNH.MOL.341367, RMNH.MOL.341369 |
| *Atlanta fragilis* | Pacific | KH1110 | 21 | -23.00 | -100.00 | 1 |  |  |  |  | AGD050-17 | Wall-Palmer et al. 2018 | RMNH.MOL.341370 |
| *Atlanta fragilis* | Pacific | ACAS | 8 | 31.24 | 173.92 | 1 |  |  |  |  | AGD031-17 | Wall-Palmer et al. 2018 | RMNH.MOL.341380 |
| *Atlanta fragilis* | Pacific | DRFT | 11 | -36.05 | -149.29 | 1 |  |  |  |  | AGD045-17 | Wall-Palmer et al. 2018 | RMNH.MOL.341365 |
| *Atlanta fragilis* | Pacific | SO255 | 41 | -34.27 | -178.87 | 1 | 1 | 1 | x |  | ATCP028-19 | New | RMNH.MOL.341362 |
| *Atlanta fragilis* | Pacific | SO255 | 143 | -32.87 | -179.78 | 1 |  | 1 |  |  | ATCP027-19 | New | RMNH.MOL.341361 |
| *Atlanta frontieri* | Indian | SN105 | 1 | 11.89 | 66.97 | 5 |  |  |  |  | AGD059-17 - AGD063-17 | Wall-Palmer et al. 2018 + New | RMNH.MOL.341389, RMNH.MOL.341390, RMNH.MOL.341392, RMNH.MOL.341385, RMNH.MOL.341387 |
| *Atlanta frontieri* | Indian | SN105 | 4 | 8.02 | 67.08 | 4 | 1 | 1 |  |  | AGD064-17 - AGD067-17 | Wall-Palmer et al. 2018 + New | RMNH.MOL.341391, RMNH.MOL.341393, RMNH.MOL.341386, RMNH.MOL.341388 |
| *Atlanta frontieri* | Indian | SN105 | 8 | 4.38 | 67.00 | 4 | 1 | 1 | x | x | AGD055-17 - AGD058-17 | Wall-Palmer et al. 2018 + New | RMNH.MOL.341381, RMNH.MOL.341382, RMNH.MOL.341383, RMNH.MOL.341384 |
| *Atlanta frontieri* | Pacific | KH1110 | 2 | -23.00 | 160.00 | 1 |  |  |  |  | AGD054-17 | New | RMNH.MOL.341398 |
| *Atlanta frontieri* | Pacific | KOK1703 | 5 | 22.65 | -157.69 | 1 | 1 |  |  |  | ATCP029-19 | New | RMNH.MOL.341394 |
| *Atlanta frontieri* | Pacific | KOK1703 | 6 | 23.52 | -156.78 | 1 | 1 |  |  |  | ATCP030-19 | New | RMNH.MOL.341395 |
| *Atlanta frontieri* | Pacific | SO255 | 73 | -28.13 | 179.02 | 2 | 2 | 2 | x |  | ATCP031-19, ATCP032-19 | New | RMNH.MOL.341396, RMNH.MOL.341397 |
| *Atlanta gaudichaudi* | Indian | VANC | 1 | -35.05 | 23.73 | 1 |  | 1 | x | x | AGD068-17 | Wall-Palmer et al. 2018 + New | RMNH.MOL.341399 |
| *Atlanta gibbosa* | Indian | SN105 | 8 | 4.38 | 67.00 | 2 | 1 | 1 | x | x | AGD070-17 - AGD071-17 | Wall-Palmer et al. 2018 + New | RMNH.MOL.341401, RMNH.MOL.341400 |
| *Atlanta gibbosa* | Pacific | S226 | 29 | 1.48 | -160.13 | 1 | 1 |  | x |  | AGD069-17 | Wall-Palmer et al. 2018 + New | RMNH.MOL.341402 |
| *Atlanta helicinoidea A* | Atlantic | AMT24 | 6 | 31.30 | -27.73 | 3 | 1 | 1 | x |  | AGD072-17 - AGD074-17 | Wall-Palmer et al. 2018 + New | RMNH.MOL.341403, RMNH.MOL.341405, RMNH.MOL.341406 |
| *Atlanta helicinoidea A* | Atlantic | AMT24 | 7 | 27.50 | -28.89 | 1 |  |  |  |  | AGD075-17 | Wall-Palmer et al. 2018 | RMNH.MOL.341404 |
| *Atlanta helicinoidea A* | Atlantic | AMT24 | 8 | 24.06 | -29.91 | 1 |  |  |  |  | AGD076-17 | Wall-Palmer et al. 2018 | RMNH.MOL.341422 |
| *Atlanta helicinoidea A* | Atlantic | AMT24 | 13 | 7.29 | -26.49 | 2 |  |  |  |  | AGD077-17 - AGD078-17 | Wall-Palmer et al. 2018 | RMNH.MOL.341423, RMNH.MOL.341424 |
| *Atlanta helicinoidea A* | Atlantic | AMT24 | 14 | 3.80 | -25.78 | 1 |  |  |  |  | AGD079-17 | Wall-Palmer et al. 2018 | RMNH.MOL.341425 |
| *Atlanta helicinoidea A* | Atlantic | AMT24 | 16 | -3.89 | -25.03 | 3 | 2 | 2 | x | x | AGD080-17 - AGD082-17 | Wall-Palmer et al. 2018 + New | RMNH.MOL.341426, RMNH.MOL.341417, RMNH.MOL.341419 |
| *Atlanta helicinoidea A* | Atlantic | AMT24 | 20 | -18.32 | -25.09 | 1 |  |  |  |  | AGD083-17 | Wall-Palmer et al. 2018 | RMNH.MOL.341427 |
| *Atlanta helicinoidea A* | Atlantic | AMT24 | 22 | -24.46 | -25.04 | 3 |  |  |  |  | AGD084-17 - AGD086-17 | Wall-Palmer et al. 2018 | RMNH.MOL.341416, RMNH.MOL.341418, RMNH.MOL.341420 |
| *Atlanta helicinoidea A* | Atlantic | AMT24 | 25A | -34.18 | -27.21 | 2 |  |  |  |  | AGD087-17 - AGD088-17 | Wall-Palmer et al. 2018 | RMNH.MOL.341407, RMNH.MOL.341408 |
| *Atlanta helicinoidea A* | Atlantic | AMT24 | 27 | -40.12 | -30.91 | 1 | 1 | 1 | x |  | AGD089-17 | Wall-Palmer et al. 2018 + New | RMNH.MOL.341421 |
| *Atlanta helicinoidea A* | Atlantic | AMT27 | 43 | -15.96 | -25.07 | 1 | 1 | 1 |  |  | ATCP034-19 | Wall-Palmer et al. 2018 + New | RMNH.MOL.341411 |
| *Atlanta helicinoidea A* | Atlantic | AMT27 | 58 | -41.16 | -30.00 | 1 |  |  |  |  | ATCP035-19 | New | RMNH.MOL.341412 |
| *Atlanta helicinoidea A* | Indian | SN105 | 8 | 4.38 | 67.00 | 1 | 1 | 1 | x |  | AGD104-17 | Wall-Palmer et al. 2018 + New | RMNH.MOL.341409 |
| *Atlanta helicinoidea A* | Indian | VANC | 5 | -34.36 | 37.73 | 1 | 1 | 1 | x |  | AGD105-17 | Wall-Palmer et al. 2018 + New | RMNH.MOL.341415 |
| *Atlanta helicinoidea A* | Pacific | KH1110 | 15 | -23.00 | -119.27 | 2 | 1 | 1 | x |  | AGD098-17 - AGD099-17 | Wall-Palmer et al. 2018 + New | RMNH.MOL.341413, RMNH.MOL.341414 |
| *Atlanta helicinoidea A* | Pacific | SO255 | 100 | -28.52 | 179.59 | 1 | 1 | 1 | x |  | ATCP033-19 | New | RMNH.MOL.341410 |
| *Atlanta helicinoidea B* | Indian | SN105 | 8 | 4.38 | 67.00 | 3 | 2 | 2 | x | x | AGD101-17 - AGD103-17 | Wall-Palmer et al. 2018 + New | RMNH.MOL.341430, RMNH.MOL.341429, RMNH.MOL.341431 |
| *Atlanta helicinoidea B* | Indian | VANC | 17 | -18.43 | 80.92 | 1 |  |  |  |  | AGD106-17 | Wall-Palmer et al. 2018 | RMNH.MOL.341441 |
| *Atlanta helicinoidea B* | Indian | VANC | 22 | -12.86 | 94.29 | 1 | 1 | 1 |  |  | AGD107-17 | Wall-Palmer et al. 2018 + New | RMNH.MOL.341443 |
| *Atlanta helicinoidea B* | Indian | VANC | 24 | -13.21 | 104.66 | 1 | 1 | 1 | x |  | AGD108-17 | Wall-Palmer et al. 2018 + New | RMNH.MOL.341433 |
| *Atlanta helicinoidea B* | Pacific | KH1110 | 2 | -23.00 | 160.00 | 4 | 1 | 1 |  |  | AGD090-17 - AGD093-17 | Wall-Palmer et al. 2018 + New | RMNH.MOL.341444, RMNH.MOL.341434, RMNH.MOL.341436, RMNH.MOL.341438 |
| *Atlanta helicinoidea B* | Pacific | KH1110 | 5 | -23.00 | 180.01 | 2 |  |  |  |  | AGD094-17 - AGD095-17 | Wall-Palmer et al. 2018 | RMNH.MOL.341439, RMNH.MOL.341442 |
| *Atlanta helicinoidea B* | Pacific | KH1110 | 8 | -22.79 | -158.10 | 2 | 1 | 1 |  |  | AGD096-17 - AGD097-17 | Wall-Palmer et al. 2018 + New | RMNH.MOL.341435, RMNH.MOL.341437 |
| *Atlanta helicinoidea B* | Pacific | S226 | 9 | 13.87 | -159.12 | 1 | 1 | 1 | x |  | AGD100-17 | Wall-Palmer et al. 2018 + New | RMNH.MOL.341440 |
| *Atlanta helicinoidea B* | Pacific | SO255 | 57 | -29.95 | -178.73 | 1 | 1 | 1 | x |  | ATCP037-19 | New | RMNH.MOL.341432 |
| *Atlanta inclinata* | Atlantic | AMT24 | 12 | 10.78 | -27.21 | 1 |  |  |  |  | AGD109-17 | Wall-Palmer et al. 2018 | RMNH.MOL.341481 |
| *Atlanta inclinata* | Atlantic | AMT24 | 13 | 7.29 | -26.49 | 3 |  |  |  |  | AGD110-17 - AGD112-17 | Wall-Palmer et al. 2018 | RMNH.MOL.341485, RMNH.MOL.341479, RMNH.MOL.341480 |
| *Atlanta inclinata* | Atlantic | AMT24 | 14 | 3.80 | -25.78 | 5 | 1 | 1 |  |  | AGD113-17 - AGD117-17 | Wall-Palmer et al. 2018 + New | RMNH.MOL.341476, RMNH.MOL.341482, RMNH.MOL.341484, RMNH.MOL.341478, RMNH.MOL.341483 |
| *Atlanta inclinata* | Atlantic | AMT24 | 15 | 0.08 | -25.02 | 2 | 1 | 1 | x | x | AGD118-17 - AGD119-17 (2) | Wall-Palmer et al. 2018 + New | RMNH.MOL.341464, RMNH.MOL.341466 |
| *Atlanta inclinata* | Atlantic | AMT24 | 16 | -3.89 | -25.03 | 3 |  |  |  |  | AGD120-17 - AGD122-17 | Wall-Palmer et al. 2018 | RMNH.MOL.341462, RMNH.MOL.341463, RMNH.MOL.341465 |
| *Atlanta inclinata* | Atlantic | AMT24 | 18 | -11.04 | -25.05 | 2 | 1 | 1 |  |  | AGD123-17 - AGD124-17 | Wall-Palmer et al. 2018 + New | RMNH.MOL.341460, RMNH.MOL.341461 |
| *Atlanta inclinata* | Atlantic | AMT27 | 33 | -0.72 | -24.97 | 1 |  |  |  |  | ATCP055-19 | New | RMNH.MOL.341473 |
| *Atlanta inclinata* | Atlantic | AMT27 | 35 | -3.54 | -25.01 | 2 | 1 | 2 |  |  | ATCP056-19 - ATCP057-19 | New | RMNH.MOL.341474, RMNH.MOL.341475 |
| *Atlanta inclinata* | Atlantic | AMT27 | 37 | -6.87 | -25.04 | 2 |  | 1 |  |  | ATCP053-19, ATCP054-19 | New | RMNH.MOL.341471, RMNH.MOL.341472 |
| *Atlanta inclinata* | Indian | SN105 | 4 | 8.02 | 67.08 | 2 | 1 |  |  |  | AGD126-17 - AGD127-17 | Wall-Palmer et al. 2018 + New | RMNH.MOL.341467, RMNH.MOL.341470 |
| *Atlanta inclinata* | Indian | SN105 | 19 | -2.95 | 66.99 | 2 | 1 | 1 | x |  | AGD128-17 - AGD129-17 | Wall-Palmer et al. 2018 + New | RMNH.MOL.341468, RMNH.MOL.341469 |
| *Atlanta inclinata* | Pacific | S226 | 45 | 9.47 | -154.42 | 1 | 1 | 1 | x |  | AGD125-17 | Wall-Palmer et al. 2018 + New | RMNH.MOL.341477 |
| *Atlanta inflata* | Indian | SN105 | 1 | 11.89 | 66.97 | 2 |  |  |  |  | AGD137-17 - AGD138-17 | Wall-Palmer et al. 2018 | RMNH.MOL.341494, RMNH.MOL.341495 |
| *Atlanta inflata* | Indian | SN105 | 8 | 4.38 | 67.00 | 3 | 1 | 1 |  |  | AGD139-17 - AGD141-17 | Wall-Palmer et al. 2018 + New | RMNH.MOL.341487, RMNH.MOL.341488, RMNH.MOL.341489 |
| *Atlanta inflata* | Indian | SN105 | 19 | -2.95 | 66.99 | 5 | 1 | 1 | x |  | AGD142-17 - AGD146-17 | Wall-Palmer et al. 2018 + New | RMNH.MOL.341492, RMNH.MOL.341493, RMNH.MOL.341486, RMNH.MOL.341490, RMNH.MOL.341491 |
| *Atlanta inflata* | Pacific | KH1110 | 2 | -23.00 | 160.00 | 2 | 2 | 1 |  |  | AGD130-17 - AGD131-17 | Wall-Palmer et al. 2018 + New | RMNH.MOL.341502, RMNH.MOL.341503 |
| *Atlanta inflata* | Pacific | KH1110 | 5 | -23.00 | 180.01 | 4 | 1 | 1 |  |  | AGD132-17 - AGD135-17 | Wall-Palmer et al. 2018 + New | RMNH.MOL.341499, RMNH.MOL.341504, RMNH.MOL.341500, RMNH.MOL.341501 |
| *Atlanta inflata* | Pacific | KH1110 | 8 | -22.79 | -158.10 | 1 | 1 | 1 |  |  | AGD136-17 | Wall-Palmer et al. 2018 + New | RMNH.MOL.341505 |
| *Atlanta inflata* | Pacific | KOK1703 | 7 | 23.62 | -157.61 |  | 1 |  |  |  | ATCP180-19 | New | RMNH.MOL.341496 |
| *Atlanta inflata* | Pacific | SO255 | 57 | -29.95 | -178.73 | 1 | 1 | 1 | x | x | ATCP058-19 | New | RMNH.MOL.341497 |
| *Atlanta inflata* | Pacific | SO255 | 100 | -28.52 | 179.59 | 1 | 1 | 1 |  |  | ATCP059-19 | New | RMNH.MOL.341498 |
| *Atlanta lesueurii* | Atlantic | AMT24 | 11 | 14.21 | -27.93 | 2 |  |  |  |  | AGD147-17 - AGD148-17 | Wall-Palmer et al. 2018 | RMNH.MOL.341507, RMNH.MOL.341508 |
| *Atlanta lesueurii* | Atlantic | AMT24 | 15 | 0.08 | -25.02 | 1 | 1 | 1 | x |  | AGD149-17 | Wall-Palmer et al. 2018 + New | RMNH.MOL.341506 |
| *Atlanta lesueurii* | Atlantic | AMT24 | 16 | -3.89 | -25.03 | 4 | 3 | 2 |  |  | AGD150-17 - AGD153-17 | Wall-Palmer et al. 2018 + New | RMNH.MOL.341515, RMNH.MOL.341516, RMNH.MOL.341517, RMNH.MOL.341514 |
| *Atlanta lesueurii* | Atlantic | AMT27 | 21 | 16.56 | -29.10 | 1 | 1 | 1 |  |  | ATCP060-19 | New | RMNH.MOL.341512 |
| *Atlanta lesueurii* | Atlantic | AMT27 | 23 | 13.15 | -28.25 | 1 |  | 1 |  |  | ATCP061-19 | New | RMNH.MOL.341513 |
| *Atlanta lesueurii* | Indian | SN105 | 8 | 4.38 | 67.00 | 1 | 1 | 1 |  |  | AGD154-17 | Wall-Palmer et al. 2018 + New | RMNH.MOL.341511 |
| *Atlanta lesueurii* | Indian | SN105 | 19 | -2.95 | 66.99 | 2 | 1 | 1 | x | x | AGD155-17 - AGD156-17 | Wall-Palmer et al. 2018 + New | RMNH.MOL.341509, RMNH.MOL.341510 |
| *Atlanta meteori A* | Indian | SN105 | 1 | 11.89 | 66.97 | 2 |  |  |  |  | AGD162-17 - AGD163-17 | Wall-Palmer et al. 2018 | RMNH.MOL.341523, RMNH.MOL.341524 |
| *Atlanta meteori A* | Indian | SN105 | 4 | 8.02 | 67.08 | 4 |  |  |  |  | AGD164-17 - AGD167-17 | Wall-Palmer et al. 2018 | RMNH.MOL.341527, RMNH.MOL.341525, RMNH.MOL.341529, RMNH.MOL.341521 |
| *Atlanta meteori A* | Indian | SN105 | 8 | 4.38 | 67.00 | 2 | 1 | 1 |  |  | AGD168-17 - AGD169-17 | Wall-Palmer et al. 2018 + New | RMNH.MOL.341526, RMNH.MOL.341528 |
| *Atlanta meteori A* | Indian | SN105 | 19 | -2.95 | 66.99 | 4 | 2 | 2 | x | x | AGD170-17 - AGD173-17 | Wall-Palmer et al. 2018 + New | RMNH.MOL.341519, RMNH.MOL.341520, RMNH.MOL.341522, RMNH.MOL.341518 |
| *Atlanta meteori A* | Pacific | KH1110 | 8 | -22.79 | -158.10 | 1 | 1 | 1 | x |  | AGD161-17 | Wall-Palmer et al. 2018 + New | RMNH.MOL.341532 |
| *Atlanta meteori A* | Pacific | KOK1703 | 3 | 22.65 | -157.69 | 1 | 2 |  |  |  | ATCP181-19, ATCP062-19 | New | RMNH.MOL.341530, RMNH.MOL.341531 |
| *Atlanta meteori B* | Atlantic | AMT24 | 8 | 24.06 | -29.91 | 2 | 1 | 1 |  |  | AGD157-17 - AGD158-17 | Wall-Palmer et al. 2018 + New | RMNH.MOL.341537, RMNH.MOL.341538 |
| *Atlanta meteori B* | Atlantic | AMT24 | 23 | -27.76 | -25.01 | 2 | 1 | 2 | x | x | AGD159-17 - AGD160-17 | Wall-Palmer et al. 2018 + New | RMNH.MOL.341533, RMNH.MOL.341534 |
| *Atlanta meteori B* | Atlantic | AMT27 | 9 | 35.30 | -26.28 | 1 | 1 | 1 | x |  | ATCP063-19 | New | RMNH.MOL.341535 |
| *Atlanta meteori B* | Atlantic | AMT27 | 51 | -30.22 | -25.78 | 1 | 1 |  |  |  | ATCP064-19 | New | RMNH.MOL.341536 |
| *Atlanta oligogyra A* | Indian | SN105 | 1 | 11.89 | 66.97 | 4 |  |  |  |  | AGD186-17 - AGD188-17, AGD190-17 | Wall-Palmer et al. 2018 | RMNH.MOL.341546, RMNH.MOL.341547, RMNH.MOL.341549, RMNH.MOL.341542 |
| *Atlanta oligogyra A* | Indian | SN105 | 4 | 8.02 | 67.08 | 4 | 1 | 1 | x |  | AGD192-17, AGD194-17 - AGD196-17 | Wall-Palmer et al. 2018 + New | RMNH.MOL.341551, RMNH.MOL.341543, RMNH.MOL.341544, RMNH.MOL.341540 |
| *Atlanta oligogyra A* | Indian | SN105 | 8 | 4.38 | 67.00 | 3 |  |  |  |  | AGD197-17 - AGD198-17, AGD200-17 | Wall-Palmer et al. 2018 | RMNH.MOL.341545, RMNH.MOL.341548, RMNH.MOL.341550 |
| *Atlanta oligogyra A* | Indian | SN105 | 19 | -2.95 | 66.99 | 2 | 1 | 1 |  |  | AGD201-17 - AGD202-17 | Wall-Palmer et al. 2018 + New | RMNH.MOL.341539, RMNH.MOL.341541 |
| *Atlanta oligogyra A* | Indian | VANC | 2 | -35.07 | 24.50 | 1 | 1 | 1 | x |  | AGD207-17 | Wall-Palmer et al. 2018 + New | RMNH.MOL.341555 |
| *Atlanta oligogyra A* | Indian | PGO | 119 | 8.97 | 69.74 | 1 | 1 |  |  |  | AGD183-17 | Wall-Palmer et al. 2018 + New | RMNH.MOL.341556 |
| *Atlanta oligogyra A* | Pacific | KH1110 | 5 | -23.00 | 180.01 | 1 |  |  |  |  | AGD181-17 | Wall-Palmer et al. 2018 | RMNH.MOL.341552 |
| *Atlanta oligogyra A* | Pacific | KH1110 | 18 | -30.00 | -107.00 | 1 | 1 | 1 | x | x | AGD182-17 | Wall-Palmer et al. 2018 + New | RMNH.MOL.341554 |
| *Atlanta oligogyra A* | Pacific | S226 | 10 | 13.08 | -159.34 | 1 | 1 | 1 | x |  | AGD184-17 | Wall-Palmer et al. 2018 + New | RMNH.MOL.341553 |
| *Atlanta oligogyra B* | Indian | SN105 | 1 | 11.89 | 66.97 | 2 | 1 | 1 | x | x | AGD185-17, AGD189-17 | Wall-Palmer et al. 2018 + New | RMNH.MOL.341558, RMNH.MOL.341560 |
| *Atlanta oligogyra B* | Indian | SN105 | 4 | 8.02 | 67.08 | 2 | 1 | 1 |  |  | AGD191-17, AGD193-17 | Wall-Palmer et al. 2018 + New | RMNH.MOL.341561, RMNH.MOL.341557 |
| *Atlanta oligogyra B* | Indian | SN105 | 8 | 4.38 | 67.00 | 1 |  |  |  |  | AGD199-17 | Wall-Palmer et al. 2018 | RMNH.MOL.341559 |
| *Atlanta oligogyra B* | Indian | VANC | 1 | -35.05 | 23.73 | 3 | 1 |  |  |  | AGD203-17 - AGD205-17 | Wall-Palmer et al. 2018 + New | RMNH.MOL.341566, RMNH.MOL.341568, RMNH.MOL.341569 |
| *Atlanta oligogyra B* | Indian | VANC | 2 | -35.07 | 24.50 | 1 | 1 | 1 | x |  | AGD206-17 | Wall-Palmer et al. 2018 + New | RMNH.MOL.341571 |
| *Atlanta oligogyra B* | Pacific | KH1110 | 2 | -23.00 | 160.00 | 3 | 1 | 1 | x |  | AGD175-17 - AGD177-17 | Wall-Palmer et al. 2018 + New | RMNH.MOL.341572, RMNH.MOL.341573, RMNH.MOL.341564 |
| *Atlanta oligogyra B* | Pacific | KH1110 | 5 | -23.00 | 180.01 | 3 | 1 | 1 |  |  | AGD178-17 - AGD180-17 | Wall-Palmer et al. 2018 + New | RMNH.MOL.341570, RMNH.MOL.341565, RMNH.MOL.341567 |
| *Atlanta oligogyra B* | Pacific | KOK1703 | 6 | 23.52 | -156.78 | 1 | 1 |  |  |  | ATCP065-19 | New | RMNH.MOL.341562 |
| *Atlanta oligogyra B* | Pacific | SO255 | 73 | -28.13 | 179.02 | 1 |  |  |  |  | ATCP066-19 | New | RMNH.MOL.341563 |
| *Atlanta oligogyra C* | Atlantic | AMT24 | 19 | -14.66 | -25.07 | 1 |  |  |  |  | AGD174-17 | Wall-Palmer et al. 2018 | RMNH.MOL.341574 |
| *Atlanta oligogyra C* | Atlantic | AMT27 | 17 | 23.36 | -29.22 | 1 | 1 | 1 | x | x | ATCP068-19 | New | RMNH.MOL.341576 |
| *Atlanta oligogyra C* | Atlantic | AMT27 | 33 | -0.72 | -24.97 | 1 | 1 |  |  |  | ATCP067-19 | New | RMNH.MOL.341575 |
| *Atlanta peronii A* | Atlantic | AMT24 | 6 | 31.30 | -27.73 | 1 | 1 | 1 | x | x | AGD210-17 | Wall-Palmer et al. 2018 + New | RMNH.MOL.341592 |
| *Atlanta peronii A* | Atlantic | AMT24 | 8 | 24.06 | -29.91 | 1 | 1 | 1 |  |  | AGD211-17 | Wall-Palmer et al. 2018 + New | RMNH.MOL.341594 |
| *Atlanta peronii A* | Atlantic | AMT24 | 18 | -11.04 | -25.05 | 1 |  |  |  |  | AGD222-17 | Wall-Palmer et al. 2018 | RMNH.MOL.341578 |
| *Atlanta peronii A* | Atlantic | AMT24 | 21 | -20.86 | -25.08 | 2 |  |  |  |  | AGD226-17 - AGD227-17 | Wall-Palmer et al. 2018 | RMNH.MOL.341591, RMNH.MOL.341593 |
| *Atlanta peronii A* | Atlantic | AMT24 | 22 | -24.46 | -25.04 | 2 | 2 | 2 |  |  | AGD228-17 - AGD229-17 | Wall-Palmer et al. 2018 + New | RMNH.MOL.341589, RMNH.MOL.341590 |
| *Atlanta peronii A* | Atlantic | AMT24 | 23 | -27.76 | -25.01 | 3 | 1 | 1 |  |  | AGD230-17 - AGD232-17 | Wall-Palmer et al. 2018 + New | RMNH.MOL.341595, RMNH.MOL.341596, RMNH.MOL.341598 |
| *Atlanta peronii A* | Atlantic | AMT24 | 25 | -34.18 | -27.22 | 1 |  |  |  |  | AGD233-17 | Wall-Palmer et al. 2018 | RMNH.MOL.341577 |
| *Atlanta peronii A* | Atlantic | AMT24 | 27 | -40.12 | -30.91 | 1 | 1 | 1 | x |  | AGD234-17 | Wall-Palmer et al. 2018 + New | RMNH.MOL.341588 |
| *Atlanta peronii A* | Indian | SN105 | 19 | -2.95 | 66.99 | 4 | 2 | 2 | x |  | AGD241-17 - AGD244-17 | Wall-Palmer et al. 2018 + New | RMNH.MOL.341579, RMNH.MOL.341580, RMNH.MOL.341581, RMNH.MOL.341582 |
| *Atlanta peronii A* | Pacific | ACAS | 2 | 28.21 | -162.14 | 1 |  | 1 |  |  | AGD208-17 | Wall-Palmer et al. 2018 + New | RMNH.MOL.341597 |
| *Atlanta peronii A* | Pacific | KOK1703 | 1 | 22.91 | -157.72 | 1 | 1 |  |  |  | ATCP070-19 | New | RMNH.MOL.341584 |
| *Atlanta peronii A* | Pacific | KOK1703 | 3 | 22.65 | -157.69 | 1 | 1 | 1 | x |  | ATCP069-19 | New | RMNH.MOL.341583 |
| *Atlanta peronii A* | Pacific | SO255 | 13 | -34.54 | 178.51 | 1 |  |  |  |  | ATCP073-19 | New | RMNH.MOL.341587 |
| *Atlanta peronii A* | Pacific | SO255 | 80 | -29.10 | -179.72 | 1 |  | 1 | x |  | ATCP071-19 | New | RMNH.MOL.341585 |
| *Atlanta peronii A* | Pacific | SO255 | 143 | -32.87 | -179.78 | 1 |  | 1 |  |  | ATCP072-19 | New | RMNH.MOL.341586 |
| *Atlanta peronii B* | Atlantic | AMT22 | 27 | 17.70 | -36.46 | 1 |  |  |  |  | AGD209-17 | Wall-Palmer et al. 2018 | RMNH.MOL.341601 |
| *Atlanta peronii B* | Atlantic | AMT24 | 8 | 24.06 | -29.91 | 1 |  |  |  |  | AGD212-17 | Wall-Palmer et al. 2018 | RMNH.MOL.341609 |
| *Atlanta peronii B* | Atlantic | AMT24 | 9 | 20.45 | -29.27 | 4 | 2 | 2 | x |  | AGD215-17 - AGD218-17 | Wall-Palmer et al. 2018 + New | RMNH.MOL.341599, RMNH.MOL.341600, RMNH.MOL.341610, RMNH.MOL.341611 |
| *Atlanta peronii B* | Atlantic | AMT27 | 9 | 35.30 | -26.28 | 1 |  |  |  |  | ATCP076-19 | New | RMNH.MOL.341604 |
| *Atlanta peronii B* | Indian | VANC | 15 | -21.04 | 75.14 | 1 |  |  |  |  | AGD245-17 | Wall-Palmer et al. 2018 | RMNH.MOL.341606 |
| *Atlanta peronii B* | Pacific | DRFT | 14 | -38.32 | -161.14 | 2 | 2 | 1 | x | x | AGD235-17 - AGD236-17 | Wall-Palmer et al. 2018 + New | RMNH.MOL.341608, RMNH.MOL.341607 |
| *Atlanta peronii B* | Pacific | KH1110 | 18 | -30.00 | -107.00 | 1 |  |  |  |  | AGD237-17 | Wall-Palmer et al. 2018 | RMNH.MOL.341612 |
| *Atlanta peronii B* | Pacific | KOK1703 | 7 | 23.62 | -157.61 | 1 | 1 | 1 | x |  | ATCP074-19 | New | RMNH.MOL.341602 |
| *Atlanta peronii B* | Pacific | SO255 | 57 | -29.95 | -178.73 | 1 |  | 1 |  |  | ATCP077-19 | New | RMNH.MOL.341605 |
| *Atlanta peronii B* | Pacific | SO255 | 100 | -28.52 | 179.59 | 1 |  |  |  |  | ATCP075-19 | New | RMNH.MOL.341603 |
| *Atlanta peronii C1* | Atlantic | AMT24 | 9 | 20.45 | -29.27 | 2 | 1 | 1 | x |  | AGD213-17, AGD214-17 | Wall-Palmer et al. 2018 + New | RMNH.MOL.341616, RMNH.MOL.341623 |
| *Atlanta peronii C1* | Atlantic | AMT24 | 16 | -3.89 | -25.03 | 3 | 2 | 2 |  |  | AGD219-17 - AGD221-17 | Wall-Palmer et al. 2018 + New | RMNH.MOL.341622, RMNH.MOL.341620, RMNH.MOL.341621 |
| *Atlanta peronii C1* | Atlantic | AMT24 | 18 | -11.04 | -25.05 | 3 | 2 | 2 | x |  | AGD223-17 - AGD225-17 | Wall-Palmer et al. 2018 + New | RMNH.MOL.341614, RMNH.MOL.341615, RMNH.MOL.341613 |
| *Atlanta peronii C2* | Pacific | KH1110 | 21 | -23.00 | -100.00 | 3 | 2 | 2 | x | x | AGD238-17 - AGD240-17 | Wall-Palmer et al. 2018 + New | RMNH.MOL.341624, RMNH.MOL.341625, RMNH.MOL.341626 |
| *Atlanta peronii C2* | Pacific | SO255 | 73 | -28.13 | 179.02 | 1 |  | 1 |  |  | ATCP080-19 | New | RMNH.MOL.341619 |
| *Atlanta peronii C2* | Pacific | SO255 | 80 | -29.10 | -179.72 | 2 | 2 | 2 | x |  | ATCP078-19, ATCP079-19 | New | RMNH.MOL.341617, RMNH.MOL.341618 |
| *Atlanta plana* | Indian | SN105 | 4 | 8.02 | 67.08 | 3 | 1 | 1 |  |  | AGD246-17 - AGD248-17 | Wall-Palmer et al. 2018 + New | RMNH.MOL.341631, RMNH.MOL.341632, RMNH.MOL.341633 |
| *Atlanta plana* | Indian | SN105 | 8 | 4.38 | 67.00 | 1 |  |  |  |  | AGD257-17 | Wall-Palmer et al. 2018 | RMNH.MOL.341627 |
| *Atlanta plana* | Indian | SN105 | 19 | -2.95 | 66.99 | 3 | 1 | 1 | x |  | AGD258-17 - AGD260-17 | Wall-Palmer et al. 2018 + New | RMNH.MOL.341628, RMNH.MOL.341629, RMNH.MOL.341630 |
| *Atlanta plana* | Indian | VANC | 1 | -35.05 | 23.73 | 1 |  |  |  |  | AGD261-17 | Wall-Palmer et al. 2018 | RMNH.MOL.341640 |
| *Atlanta plana* | Indian | VANC | 2 | -35.07 | 24.50 | 1 |  |  |  |  | AGD262-17 | Wall-Palmer et al. 2018 | RMNH.MOL.341643 |
| *Atlanta plana* | Pacific | KM1109 | 9 | 21.333 | -158.354 | 1 | 1 | 1 | x | x | AGD253-17 | Wall-Palmer et al. 2018 + New | RMNH.MOL.341638 |
| *Atlanta plana* | Pacific | KM1109 | 10 | 21.414 | -158.343 | 1 |  |  |  |  | AGD254-17 | Wall-Palmer et al. 2018 | RMNH.MOL.341639 |
| *Atlanta plana* | Pacific | KH1110 | 8 | -22.79 | -158.10 | 4 |  |  |  |  | AGD249-17 - AGD252-17 | Wall-Palmer et al. 2018 | RMNH.MOL.341637, RMNH.MOL.341644, RMNH.MOL.341645, RMNH.MOL.341646 |
| *Atlanta plana* | Pacific | S226 | 10 | 13.083 | -159.343 | 2 |  |  |  |  | AGD255-17 - AGD256-17 | Wall-Palmer et al. 2018 | RMNH.MOL.341641, RMNH.MOL.341642 |
| *Atlanta plana* | Pacific | KOK1703 | 1 | 22.91 | -157.72 | 1 |  |  |  |  | ATCP081-19 | New | RMNH.MOL.341634 |
| *Atlanta plana* | Pacific | SO255 | 80 | -29.10 | -179.72 | 2 |  | 1 |  |  | ATCP082-19, ATCP083-19 | New | RMNH.MOL.341635, RMNH.MOL.341636 |
| *Atlanta rosea A* | Indian | VANC | 15 | -21.04 | 75.14 | 1 | 1 |  |  |  | AGD285-17 | Wall-Palmer et al. 2018 + New | RMNH.MOL.341648 |
| *Atlanta rosea A* | Indian | VANC | 17 | -18.43 | 80.92 | 3 | 2 | 2 | x |  | AGD286-17 - AGD288-17 | Wall-Palmer et al. 2018 + New | RMNH.MOL.341649, RMNH.MOL.341650, RMNH.MOL.341651 |
| *Atlanta rosea A* | Pacific | KH1110 | 5 | -23.00 | 180.01 | 2 | 2 | 2 | x | x | AGD275-17, AGD277-17 | Wall-Palmer et al. 2018 + New | RMNH.MOL.341652, RMNH.MOL.341653 |
| *Atlanta rosea A* | Pacific | KH1110 | 15 | -23.00 | -119.27 | 2 | 2 |  | x |  | AGD278-17 - AGD279-17 | Wall-Palmer et al. 2018 + New | RMNH.MOL.341660, RMNH.MOL.341661 |
| *Atlanta rosea A* | Pacific | KH1110 | 21 | -23.00 | -100.00 | 3 | 1 |  |  |  | AGD282-17 - AGD284-17 | Wall-Palmer et al. 2018 + New | RMNH.MOL.341657, RMNH.MOL.341658, RMNH.MOL.341659 |
| *Atlanta rosea A* | Pacific | KOK1703 | 5 | 22.65 | -157.69 |  | 1 |  |  |  | ATCP182-19 | New | RMNH.MOL.341654 |
| *Atlanta rosea A* | Pacific | SO255 | 100 | -28.52 | 179.59 | 1 | 1 | 1 |  |  | ATCP085-19 | New | RMNH.MOL.341655 |
| *Atlanta rosea A* | Pacific | SO255 | 143 | -32.87 | -179.78 | 2 | 1 | 2 | x |  | ATCP084-19, ATCP086-19 | New | RMNH.MOL.341647, RMNH.MOL.341656 |
| *Atlanta rosea B* | Atlantic | AMT24 | 6 | 31.30 | -27.73 | 4 | 1 | 1 | x |  | AGD263-17 - AGD266-17 | Wall-Palmer et al. 2018 + New | RMNH.MOL.341669, RMNH.MOL.341665, RMNH.MOL.341666, RMNH.MOL.341667 |
| *Atlanta rosea B* | Atlantic | AMT24 | 7 | 27.50 | -28.89 | 1 | 1 | 1 |  |  | AGD267-17 | Wall-Palmer et al. 2018 + New | RMNH.MOL.341668 |
| *Atlanta rosea B* | Atlantic | AMT24 | 19 | -14.66 | -25.07 | 3 | 2 | 2 | x | x | AGD269-17 - AGD271-17 | Wall-Palmer et al. 2018 + New | RMNH.MOL.341662, RMNH.MOL.341663, RMNH.MOL.341664 |
| *Atlanta rosea C* | Atlantic | AMT24 | 8 | 24.06 | -29.91 | 1 | 1 | 1 | x |  | AGD268-17 | Wall-Palmer et al. 2018 + New | RMNH.MOL.341675 |
| *Atlanta rosea C* | Atlantic | AMT24 | 19 | -14.66 | -25.07 | 1 | 1 | 1 | x |  | AGD272-17 | Wall-Palmer et al. 2018 + New | RMNH.MOL.341671 |
| *Atlanta rosea C* | Atlantic | AMT24 | 21 | -20.86 | -25.08 | 1 |  |  |  |  | AGD273-17 | Wall-Palmer et al. 2018 | RMNH.MOL.341670 |
| *Atlanta rosea C* | Atlantic | AMT24 | 27 | -40.12 | -30.91 | 1 | 1 | 1 | x | x | AGD274-17 | Wall-Palmer et al. 2018 + New | RMNH.MOL.341674 |
| *Atlanta rosea C* | Atlantic | AMT27 | 17 | 23.36 | -29.22 | 1 | 1 | 1 |  |  | ATCP088-19 | New | RMNH.MOL.341673 |
| *Atlanta rosea C* | Pacific | KH1110 | 5 | -23.00 | 180.01 | 1 | 1 | 1 | x |  | AGD276-17 | Wall-Palmer et al. 2018 + New | RMNH.MOL.341676 |
| *Atlanta rosea C* | Pacific | KH1110 | 18 | -30.00 | -107.00 | 2 | 2 | 2 | x |  | AGD280-17 - AGD281-17 | Wall-Palmer et al. 2018 + New | RMNH.MOL.341677, RMNH.MOL.341678 |
| *Atlanta rosea C* | Pacific | SO255 | 73 | -28.13 | 179.02 | 1 | 1 | 1 | x |  | ATCP087-19 | New | RMNH.MOL.341672 |
| *Atlanta selvagensis* | Atlantic | AMT24 | 5 | 34.75 | -26.62 | 2 | 2 |  |  |  | KX343194 (GB), ATCP368-19, AGD289-17 | Wall-Palmer et al. 2016, 2018 + New | RMNH.MOL.341688, RMNH.MOL.341679 |
| *Atlanta selvagensis* | Atlantic | AMT24 | 6 | 31.30 | -27.73 | 9 | 1 | 1 | x | x | KX343195 - KX343197 (GB), AGD290-17 - AGD295-17, ATCP369-19 | Wall-Palmer et al. 2016, 2018 + New | RMNH.MOL.341681, RMNH.MOL.341683, RMNH.MOL.341684, RMNH.MOL.341685, RMNH.MOL.341686, RMNH.MOL.341687, RMNH.MOL.341695, RMNH.MOL.341697, RMNH.MOL.341711 |
| *Atlanta selvagensis* | Atlantic | AMT24 | 7 | 27.50 | -28.89 | 3 | 1 | 1 |  |  | AGD296-17 - AGD298-17 | Wall-Palmer et al. 2018 + New | RMNH.MOL.341699, RMNH.MOL.341701, RMNH.MOL.341703 |
| *Atlanta selvagensis* | Atlantic | AMT24 | 8 | 24.06 | -29.91 | 3 | 1 | 1 |  |  | AGD299-17 - AGD301-17 | Wall-Palmer et al. 2018 + New | RMNH.MOL.341705, RMNH.MOL.341707, RMNH.MOL.341694 |
| *Atlanta selvagensis* | Atlantic | AMT24 | 9 | 20.45 | -29.27 | 3 | 1 | 1 |  |  | AGD302-17 - AGD304-17 | Wall-Palmer et al. 2018 + New | RMNH.MOL.341696, RMNH.MOL.341698, RMNH.MOL.341700 |
| *Atlanta selvagensis* | Atlantic | AMT24 | 10 | 17.82 | -28.70 | 3 | 1 | 2 |  |  | AGD305-17 - AGD307-17 | Wall-Palmer et al. 2018 + New | RMNH.MOL.341709, RMNH.MOL.341710, RMNH.MOL.341712 |
| *Atlanta selvagensis* | Atlantic | AMT24 | 14 | 3.80 | -25.78 | 3 |  | 1 |  |  | KX343198 (GB), AGD308-17 - AGD309-17 | Wall-Palmer et al. 2016, 2018 + New | RMNH.MOL.341680, RMNH.MOL.341689, RMNH.MOL.341682 |
| *Atlanta selvagensis* | Atlantic | AMT24 | 15 | 0.08 | -25.02 | 4 |  |  |  |  | AGD310-17 - AGD313-17 | Wall-Palmer et al. 2018 | RMNH.MOL.341693, RMNH.MOL.341708, RMNH.MOL.341713, RMNH.MOL.341714 |
| *Atlanta selvagensis* | Atlantic | AMT24 | 16 | -3.89 | -25.03 | 4 | 2 | 2 | x |  | AGD314-17 - AGD317-17 | Wall-Palmer et al. 2018 + New | RMNH.MOL.341692, RMNH.MOL.341702, RMNH.MOL.341704, RMNH.MOL.341706 |
| *Atlanta selvagensis* | Atlantic | AMT27 | 37 | -6.87 | -25.04 | 2 | 1 | 2 | x |  | ATCP089-19, ATCP090-19 | New | RMNH.MOL.341690, RMNH.MOL.341691 |
| *Atlanta tokiokai* | Atlantic | AMT24 | 7 | 27.50 | -28.89 | 1 | 1 | 1 | x | x | AGD319-17 | Wall-Palmer et al. 2018 + New | RMNH.MOL.341757 |
| *Atlanta tokiokai* | Atlantic | AMT24 | 8 | 24.06 | -29.91 | 3 | 1 | 1 |  |  | AGD320-17 - AGD322-17 | Wall-Palmer et al. 2018 + New | RMNH.MOL.341758, RMNH.MOL.341759, RMNH.MOL.341760 |
| *Atlanta tokiokai* | Atlantic | AMT24 | 9 | 20.45 | -29.27 | 4 | 1 | 2 |  |  | AGD323-17 - AGD326-17 | Wall-Palmer et al. 2018 + New | RMNH.MOL.341715, RMNH.MOL.341716, RMNH.MOL.341717, RMNH.MOL.341718 |
| *Atlanta tokiokai* | Atlantic | AMT27 | 43 | -15.96 | -25.07 | 1 | 1 |  |  |  | ATCP093-19 | New | RMNH.MOL.341746 |
| *Atlanta tokiokai* | Indian | SN105 | 1 | 11.89 | 66.97 | 9 |  |  |  |  | AGD335-17 - AGD343-17 | Wall-Palmer et al. 2018 | RMNH.MOL.341730, RMNH.MOL.341731, RMNH.MOL.341732, RMNH.MOL.341733, RMNH.MOL.341734, RMNH.MOL.341735, RMNH.MOL.341736, RMNH.MOL.341741, RMNH.MOL.341742 |
| *Atlanta tokiokai* | Indian | SN105 | 4 | 8.02 | 67.08 | 7 | 2 | 2 | x |  | AGD344-17 - AGD350-17 | Wall-Palmer et al. 2018 + New | RMNH.MOL.341721, RMNH.MOL.341723, RMNH.MOL.341737, RMNH.MOL.341738, RMNH.MOL.341739, RMNH.MOL.341740, RMNH.MOL.341743 |
| *Atlanta tokiokai* | Indian | SN105 | 8 | 4.38 | 67.00 | 5 |  |  |  |  | AGD351-17 - AGD355-17 | Wall-Palmer et al. 2018 | RMNH.MOL.341719, RMNH.MOL.341720, RMNH.MOL.341725, RMNH.MOL.341727, RMNH.MOL.341729, |
| *Atlanta tokiokai* | Indian | SN105 | 19 | -2.95 | 66.99 | 4 |  | 1 |  |  | AGD356-17 - AGD359-17 | Wall-Palmer et al. 2018 + New | RMNH.MOL.341722, RMNH.MOL.341724, RMNH.MOL.341726, RMNH.MOL.341728, |
| *Atlanta tokiokai* | Indian | VANC | 24 | -13.21 | 104.66 | 1 |  | 1 |  |  | AGD360-17 | Wall-Palmer et al. 2018 + New | RMNH.MOL.341755 |
| *Atlanta tokiokai* | Pacific | ACAS | 14 | 32.86 | 149.52 | 1 | 1 |  |  |  | AGD318-17 | Wall-Palmer et al. 2018 + New | RMNH.MOL.341756 |
| *Atlanta tokiokai* | Pacific | KH1110 | 8 | -22.79 | -158.10 | 1 |  |  |  |  | AGD327-17 | Wall-Palmer et al. 2018 | RMNH.MOL.341767 |
| *Atlanta tokiokai* | Pacific | KH1110 | 15 | -23.00 | -119.27 | 3 | 1 | 2 | x |  | AGD328-17 - AGD330-17 | Wall-Palmer et al. 2018 + New | RMNH.MOL.341761, RMNH.MOL.341763, RMNH.MOL.341764 |
| *Atlanta tokiokai* | Pacific | KH1110 | 18 | -30.00 | -107.00 | 2 |  |  |  |  | AGD331-17 - AGD332-17 | Wall-Palmer et al. 2018 | RMNH.MOL.341765, RMNH.MOL.341766 |
| *Atlanta tokiokai* | Pacific | KH1110 | 21 | -23.00 | -100.00 | 2 | 1 | 1 |  |  | AGD333-17 - AGD334-17 | Wall-Palmer et al. 2018 + New | RMNH.MOL.341768, RMNH.MOL.341762 |
| *Atlanta tokiokai* | Pacific | KOK1703 | 5 | 22.65 | -157.69 | 1 | 1 |  |  |  | ATCP091-19 | New | RMNH.MOL.341744 |
| *Atlanta tokiokai* | Pacific | SO255 | 57 | -29.95 | -178.73 | 2 | 1 | 2 | x |  | ATCP092-19, ATCP096-19 | New | RMNH.MOL.341745, RMNH.MOL.341749 |
| *Atlanta tokiokai* | Pacific | SO255 | 73 | -28.13 | 179.02 | 1 | 1 |  |  |  | ATCP098-19 | New | RMNH.MOL.341751 |
| *Atlanta tokiokai* | Pacific | SO255 | 80 | -29.10 | -179.72 | 2 | 1 | 1 |  |  | ATCP094-19, ATCP099-19 | New | RMNH.MOL.341747, RMNH.MOL.341752 |
| *Atlanta tokiokai* | Pacific | SO255 | 100 | -28.52 | 179.59 | 2 | 2 | 2 |  |  | ATCP095-19, ATCP100-19 | New | RMNH.MOL.341748, RMNH.MOL.341753 |
| *Atlanta tokiokai* | Pacific | SO255 | 143 | -32.87 | -179.78 | 2 | 1 |  |  |  | ATCP101-19, ATCP097-19 |  | RMNH.MOL.341750, RMNH.MOL.341754 |
| *Atlanta turriculata* | Indian | SN105 | 1 | 11.89 | 66.97 | 6 |  |  |  |  | AGD367-17 - AGD372-17 | Wall-Palmer et al. 2018 | RMNH.MOL.341779, RMNH.MOL.341780, RMNH.MOL.341781, RMNH.MOL.341782, RMNH.MOL.341783, RMNH.MOL.341775 |
| *Atlanta turriculata* | Indian | SN105 | 4 | 8.02 | 67.08 | 4 | 1 | 1 |  |  | AGD373-17 - AGD376-17 | Wall-Palmer et al. 2018 + New | RMNH.MOL.341784, RMNH.MOL.341785, RMNH.MOL.341776, RMNH.MOL.341774 |
| *Atlanta turriculata* | Indian | SN105 | 8 | 4.38 | 67.00 | 4 |  |  |  |  | AGD377-17 - AGD380-17 | Wall-Palmer et al. 2018 | RMNH.MOL.341786, RMNH.MOL.341777, RMNH.MOL.341778, RMNH.MOL.341769 |
| *Atlanta turriculata* | Indian | SN105 | 19 | -2.95 | 66.99 | 4 | 1 | 1 | x | x | AGD381-17 - AGD384-17 | Wall-Palmer et al. 2018 + New | RMNH.MOL.341770, RMNH.MOL.341771, RMNH.MOL.341772, RMNH.MOL.341773 |
| *Atlanta turriculata* | Pacific | KH1110 | 5 | -23.00 | 180.01 | 3 | 2 | 2 |  |  | AGD361-17 - AGD363-17 | Wall-Palmer et al. 2018 + New | RMNH.MOL.341801, RMNH.MOL.341802, RMNH.MOL.341797 |
| *Atlanta turriculata* | Pacific | KH1110 | 8 | -22.79 | -158.10 | 3 |  |  |  |  | AGD364-17 - AGD366-17 | Wall-Palmer et al. 2018 | RMNH.MOL.341798, RMNH.MOL.341799, RMNH.MOL.341800 |
| *Atlanta turriculata* | Pacific | KOK1703 | 1 | 22.91 | -157.72 | 1 | 1 | 1 | x |  | ATCP103-19 | New | RMNH.MOL.341788 |
| *Atlanta turriculata* | Pacific | KOK1703 | 3 | 22.65 | -157.69 | 1 | 1 |  |  |  | ATCP102-19 | New | RMNH.MOL.341787 |
| *Atlanta turriculata* | Pacific | KOK1703 | 5 | 22.65 | -157.69 | 1 | 1 |  |  |  | ATCP104-19 | New | RMNH.MOL.341789 |
| *Atlanta turriculata* | Pacific | SO255 | 57 | -29.95 | -178.73 | 1 | 1 | 1 | x |  | ATCP105-19 | New | RMNH.MOL.341790 |
| *Atlanta turriculata* | Pacific | SO255 | 73 | -28.13 | 179.02 | 1 |  | 1 |  |  | ATCP106-19 | New | RMNH.MOL.341791 |
| *Atlanta turriculata* | Pacific | SO255 | 80 | -29.10 | -179.72 | 4 | 3 | 4 |  |  | ATCP107-19 - ATCP110-19 | New | RMNH.MOL.341792, RMNH.MOL.341793, RMNH.MOL.341794, RMNH.MOL.341795 |
| *Atlanta turriculata* | Pacific | SO255 | 100 | -28.52 | 179.59 | 1 |  |  |  |  | ATCP111-19 | New | RMNH.MOL.341796 |
| *Oxygyrus inflatus A1* | Atlantic | AMT24 | 7 | 27.50 | -28.89 | 2 | 1 | 2 |  |  | AGD389-17 - AGD390-17 | Wall-Palmer et al. 2018 + New | RMNH.MOL.341862, RMNH.MOL.341863 |
| *Oxygyrus inflatus A1* | Atlantic | AMT24 | 9 | 20.45 | -29.27 | 1 | 1 |  |  |  | AGD391-17 | Wall-Palmer et al. 2018 + New | RMNH.MOL.341864 |
| *Oxygyrus inflatus A1* | Atlantic | AMT27 | 9 | 35.30 | -26.28 | 1 | 1 | 1 | x |  | ATCP164-19 | New | RMNH.MOL.341861 |
| *Oxygyrus inflatus A1* | Atlantic | AMT27 | 37 | -6.87 | -25.04 | 1 |  | 1 |  |  | ATCP162-19 | New | RMNH.MOL.341859 |
| *Oxygyrus inflatus A1* | Atlantic | AMT27 | 49 | -27.58 | -25.19 | 1 | 1 | 1 | x | x | ATCP163-19 | New | RMNH.MOL.341860 |
| *Oxygyrus inflatus A2* | Pacific | KH1110 | 8 | -22.79 | -158.10 | 1 | 1 | 1 |  |  | AGD399-17 | Wall-Palmer et al. 2018 + New | RMNH.MOL.341869 |
| *Oxygyrus inflatus A2* | Pacific | KH1110 | 18 | -30.00 | -107.00 | 2 | 1 | 2 | x |  | AGD403-17 - AGD404-17 | Wall-Palmer et al. 2018 + New | RMNH.MOL.341870, RMNH.MOL.341871 |
| *Oxygyrus inflatus A2* | Pacific | SO255 | 57 | -29.95 | -178.73 | 1 |  |  |  |  | ATCP166-19 | New | RMNH.MOL.341866 |
| *Oxygyrus inflatus A2* | Pacific | SO255 | 73 | -28.13 | 179.02 | 1 | 1 |  |  |  | ATCP167-19 | New | RMNH.MOL.341867 |
| *Oxygyrus inflatus A2* | Pacific | SO255 | 80 | -29.10 | -179.72 | 1 | 1 | 1 | x |  | ATCP168-19 | New | RMNH.MOL.341868 |
| *Oxygyrus inflatus A2* | Pacific | SO255 | 143 | -32.87 | -179.78 | 1 | 1 | 1 |  |  | ATCP165-19 | New | RMNH.MOL.341865 |
| *Oxygyrus inflatus B* | Atlantic | AMT24 | 14 | 3.80 | -25.78 | 3 | 2 | 1 | x | x | AGD392-17 - AGD394-17 | Wall-Palmer et al. 2018 + New | RMNH.MOL.341872, RMNH.MOL.341873, RMNH.MOL.341874 |
| *Oxygyrus inflatus B* | Atlantic | AMT24 | 18 | -11.04 | -25.05 | 3 | 1 |  |  |  | AGD395-17 - AGD397-17 | Wall-Palmer et al. 2018 + New | RMNH.MOL.341875, RMNH.MOL.341876, RMNH.MOL.341877 |
| *Oxygyrus inflatus B* | Atlantic | AMT27 | 33 | -0.72 | -24.97 | 1 | 1 |  |  |  | ATCP169-19 | New | RMNH.MOL.341878 |
| *Oxygyrus inflatus B* | Atlantic | AMT27 | 37 | -6.87 | -25.04 | 1 | 1 | 1 | x |  | ATCP170-19 | New | RMNH.MOL.341879 |
| *Oxygyrus inflatus C* | Indian | SN105 | 4 | 8.02 | 67.08 | 3 | 1 | 1 | x | x | AGD406-17 - AGD408-17 | Wall-Palmer et al. 2018 + New | RMNH.MOL.341883, RMNH.MOL.341884, RMNH.MOL.341885 |
| *Oxygyrus inflatus C* | Indian | SN105 | 8 | 4.38 | 67.00 | 3 |  |  |  |  | AGD409-17 - AGD411-17 | Wall-Palmer et al. 2018 | RMNH.MOL.341886, RMNH.MOL.341887, RMNH.MOL.341888 |
| *Oxygyrus inflatus C* | Indian | SN105 | 19 | -2.95 | 66.99 | 3 | 1 | 1 |  |  | AGD412-17 - AGD414-17 | Wall-Palmer et al. 2018 + New | RMNH.MOL.341880, RMNH.MOL.341881, RMNH.MOL.341882 |
| *Oxygyrus inflatus C* | Pacific | KM1109 | 9 | 21.33 | -158.35 | 1 | 1 | 1 | x |  | AGD405-17 | Wall-Palmer et al. 2018 + New | RMNH.MOL.341891 |
| *Oxygyrus inflatus C* | Pacific | KH1110 | 2 | -23.00 | 160.00 | 1 |  |  |  |  | AGD398-17 | Wall-Palmer et al. 2018 | RMNH.MOL.341892 |
| *Oxygyrus inflatus C* | Pacific | KH1110 | 8 | -22.79 | -158.10 | 2 | 2 | 2 | x |  | AGD401-17 - AGDF402-17 | Wall-Palmer et al. 2018 + New | RMNH.MOL.341893, RMNH.MOL.341890 |
| *Oxygyrus inflatus C* | Pacific | KOK1703 | 5 | 22.65 | -157.69 |  | 1 | 1 |  |  | ATCP183-19 | New | RMNH.MOL.341889 |
| *Protatlanta sculpta* | Atlantic | AMT24 | 9 | 20.45 | -29.27 | 3 | 1 | 1 |  |  | KU841485 - KU841487 (GB), ATCP359-19 | Wall-Palmer et al. 2016b + New | RMNH.MOL.341901, RMNH.MOL.341894, RMNH.MOL.341895 |
| *Protatlanta sculpta* | Atlantic | AMT24 | 10 | 17.82 | -28.70 | 1 |  |  |  |  | KU841488 (GB) | Wall-Palmer et al. 2016b | RMNH.MOL.341897 |
| *Protatlanta sculpta* | Atlantic | AMT24 | 13 | 7.29 | -26.49 | 4 | 2 | 2 |  |  | AGD415-17 - AGD418-17 | Wall-Palmer et al. 2018 + New | RMNH.MOL.341910, RMNH.MOL.341909, RMNH.MOL.341904, RMNH.MOL.341905 |
| *Protatlanta sculpta* | Atlantic | AMT24 | 16 | -3.89 | -25.03 | 3 | 2 | 1 |  |  | KU841489 - KU841491 (GB), ATCP360-19, ATCP361-19 | Wall-Palmer et al. 2016b + New | RMNH.MOL.341896, RMNH.MOL.341898, RMNH.MOL.341899 |
| *Protatlanta sculpta* | Atlantic | AMT24 | 20 | -18.32 | -25.09 | 3 |  |  |  |  | AGD419-17 - AGD421-17 | Wall-Palmer et al. 2018 | RMNH.MOL.341906, RMNH.MOL.341907, RMNH.MOL.341908 |
| *Protatlanta sculpta* | Atlantic | AMT24 | 25 | -34.18 | -27.22 | 1 |  |  |  |  | KU841492 (GB) | Wall-Palmer et al. 2016b | RMNH.MOL.341900 |
| *Protatlanta sculpta* | Atlantic | AMT27 | 9 | 35.30 | -26.28 | 1 | 1 | 1 | x | x | ATCP171-19 | New | RMNH.MOL.341902 |
| *Protatlanta sculpta* | Atlantic | AMT27 | 35 | -3.54 | -25.01 | 1 | 1 | 1 | x |  | ATCP172-19 | New | RMNH.MOL.341903 |
| *Protatlanta souleyeti* | Atlantic | AMT24 | 6 | 31.30 | -27.73 | 7 | 2 | 2 | x |  | KU841493 - KU841497 (GB), AGD425-17 - AGD426-17, ATCP362-19, ATCP363-19 | Wall-Palmer et al. 2016b, 2018 + New | RMNH.MOL.341916, RMNH.MOL.341917, RMNH.MOL.341920, RMNH.MOL.341911, RMNH.MOL.341912, RMNH.MOL.341933, RMNH.MOL.341935 |
| *Protatlanta souleyeti* | Atlantic | AMT24 | 18 | -11.04 | -25.05 | 1 |  |  |  |  | KU841500 (GB) | Wall-Palmer et al. 2016b | RMNH.MOL.341919 |
| *Protatlanta souleyeti* | Atlantic | AMT24 | 19 | -14.66 | -25.07 | 2 |  |  |  |  | KU841501 - KU841502 (GB) | Wall-Palmer et al. 2016b | RMNH.MOL.341913, RMNH.MOL.341914 |
| *Protatlanta souleyeti* | Atlantic | AMT24 | 20 | -18.32 | -25.09 | 3 | 1 | 1 | x |  | AGD427-17 - AGD429-17 | Wall-Palmer et al. 2018 + New | RMNH.MOL.341931, RMNH.MOL.341932, RMNH.MOL.341934 |
| *Protatlanta souleyeti* | Atlantic | AMT24 | 23 | -27.76 | -25.01 | 1 |  |  |  |  | KU841506 (GB) | Wall-Palmer et al. 2016b | RMNH.MOL.341915 |
| *Protatlanta souleyeti* | Atlantic | AMT24 | 25A | -34.18 | -27.21 | 1 |  | 1 |  |  | KU841507 (GB), ATCP391-19 | Wall-Palmer et al. 2016b + New | RMNH.MOL.341918 |
| *Protatlanta souleyeti* | Atlantic | AMT24 | 27 | -40.12 | -30.91 | 2 | 1 | 1 | x |  | AGD430-17 - AGD431-17 | Wall-Palmer et al. 2018 + New | RMNH.MOL.341936, RMNH.MOL.341937 |
| *Protatlanta souleyeti* | Atlantic | AMT27 | 9 | 35.30 | -26.28 | 1 | 1 | 1 |  |  | ATCP176-19 | New | RMNH.MOL.341926 |
| *Protatlanta souleyeti* | Atlantic | AMT27 | 17 | 23.36 | -29.22 | 1 | 1 | 1 |  |  | ATCP177-19 | New | RMNH.MOL.341928 |
| *Protatlanta souleyeti* | Indian | VANC | 2 | -35.07 | 24.50 |  |  | 1 |  |  | ATCP190-19 | New | RMNH.MOL.341930 |
| *Protatlanta souleyeti* | Pacific | ACAS | 8 | 31.24 | 173.92 | 2 | 2 | 2 | x | x | AGD422-17 - AGD423-17 | Wall-Palmer et al. 2018 + New | RMNH.MOL.341947, RMNH.MOL.341950 |
| *Protatlanta souleyeti* | Pacific | ACAS | 14 | 32.86 | 149.52 | 1 | 1 | 1 |  |  | AGD424-17 | Wall-Palmer et al. 2018 + New | RMNH.MOL.341939 |
| *Protatlanta souleyeti* | Pacific | KH1110 | 2 | -23.00 | 160.00 | 3 | 3 | 3 | x |  | AGD432-17 - AGD434-17 | Wall-Palmer et al. 2018 + New | RMNH.MOL.341941, RMNH.MOL.341943, RMNH.MOL.341945 |
| *Protatlanta souleyeti* | Pacific | KH1110 | 8 | -22.79 | -158.10 | 2 | 2 | 1 |  |  | AGD435-17 - AGD436-17 | Wall-Palmer et al. 2018 + New | RMNH.MOL.341948, RMNH.MOL.341951 |
| *Protatlanta souleyeti* | Pacific | KH1110 | 18 | -30.00 | -107.00 | 3 | 1 | 2 | x |  | AGD437-17 - AGD439-17 | Wall-Palmer et al. 2018 + New | RMNH.MOL.341938, RMNH.MOL.341940, RMNH.MOL.341942 |
| *Protatlanta souleyeti* | Pacific | KH1110 | 21 | -23.00 | -100.00 | 3 |  | 1 |  |  | AGD440-17 - AGD442-17 | Wall-Palmer et al. 2018 + New | RMNH.MOL.341944, RMNH.MOL.341946, RMNH.MOL.341949 |
| *Protatlanta souleyeti* | Pacific | KOK1703 | 3 | 22.65 | -157.69 | 1 | 1 | 1 |  |  | ATCP173-19 | New | RMNH.MOL.341921 |
| *Protatlanta souleyeti* | Pacific | SO255 | 57 | -29.95 | -178.73 |  | 1 | 1 |  |  | ATCP186-19 | New | RMNH.MOL.341929 |
| *Protatlanta souleyeti* | Pacific | SO255 | 73 | -28.13 | 179.02 | 1 | 1 | 1 |  |  | ATCP174-19 | New | RMNH.MOL.341922 |
| *Protatlanta souleyeti* | Pacific | SO255 | 80 | -29.10 | -179.72 | 1 |  | 2 |  |  | ATCP175-19, ATCP189-19 | New | RMNH.MOL.341923, RMNH.MOL.341927 |
| *Protatlanta souleyeti* | Pacific | SO255 | 100 | -28.52 | 179.59 |  | 1 | 1 |  |  | ATCP184-19 | New | RMNH.MOL.341924 |
| *Protatlanta souleyeti* | Pacific | SO255 | 143 | -32.87 | -179.78 |  | 1 |  |  |  | ATCP185-19 | New | RMNH.MOL.341925 |
| *Carinaria* sp. | Indian | SN105 | 1 | 11.89 | 66.97 | 4 | 1 | 1 |  |  | ATCP156-19, ATCP157-19, ATCP154-19, ATCP161-19 | New | RMNH.MOL.341849, RMNH.MOL.341851, RMNH.MOL.341852, RMNH.MOL.341856 |
| *Carinaria* sp. | Indian | SN105 | 4 | 8.02 | 67.08 | 3 |  |  |  |  | ATCP158-19, ATCP159-19, ATCP160-19 | New | RMNH.MOL.341853, RMNH.MOL.341854, RMNH.MOL.341855 |
| *Carinaria* sp. | Indian | SN105 | 8 | 4.38 | 67.00 | 4 |  |  |  |  | ATCP149-19, ATCP150-19, ATCP151-19, ATCP153-19 | New | RMNH.MOL.341844, RMNH.MOL.341845, RMNH.MOL.341846, RMNH.MOL.341848 |
| *Carinaria* sp. | Indian | SN105 | 19 | -2.95 | 66.99 | 4 | 2 | 3 | x | x | ATCP152-19, ATCP155-19, ATCP147-19, ATCP148-19 | New | RMNH.MOL.341842, RMNH.MOL.341843, RMNH.MOL.341847, RMNH.MOL.341850 |
