# Supplementary figures and images for: Fossil-calibrated molecular phylogeny of atlantid heteropods (Gastropoda, Pterotracheoidea)
Source: BMC Evol Biol. 2020 Sep 21;20:124. doi: 10.1186/s12862-020-01682-9 (PMC7507655; doi:10.1186/s12862-020-01682-9)

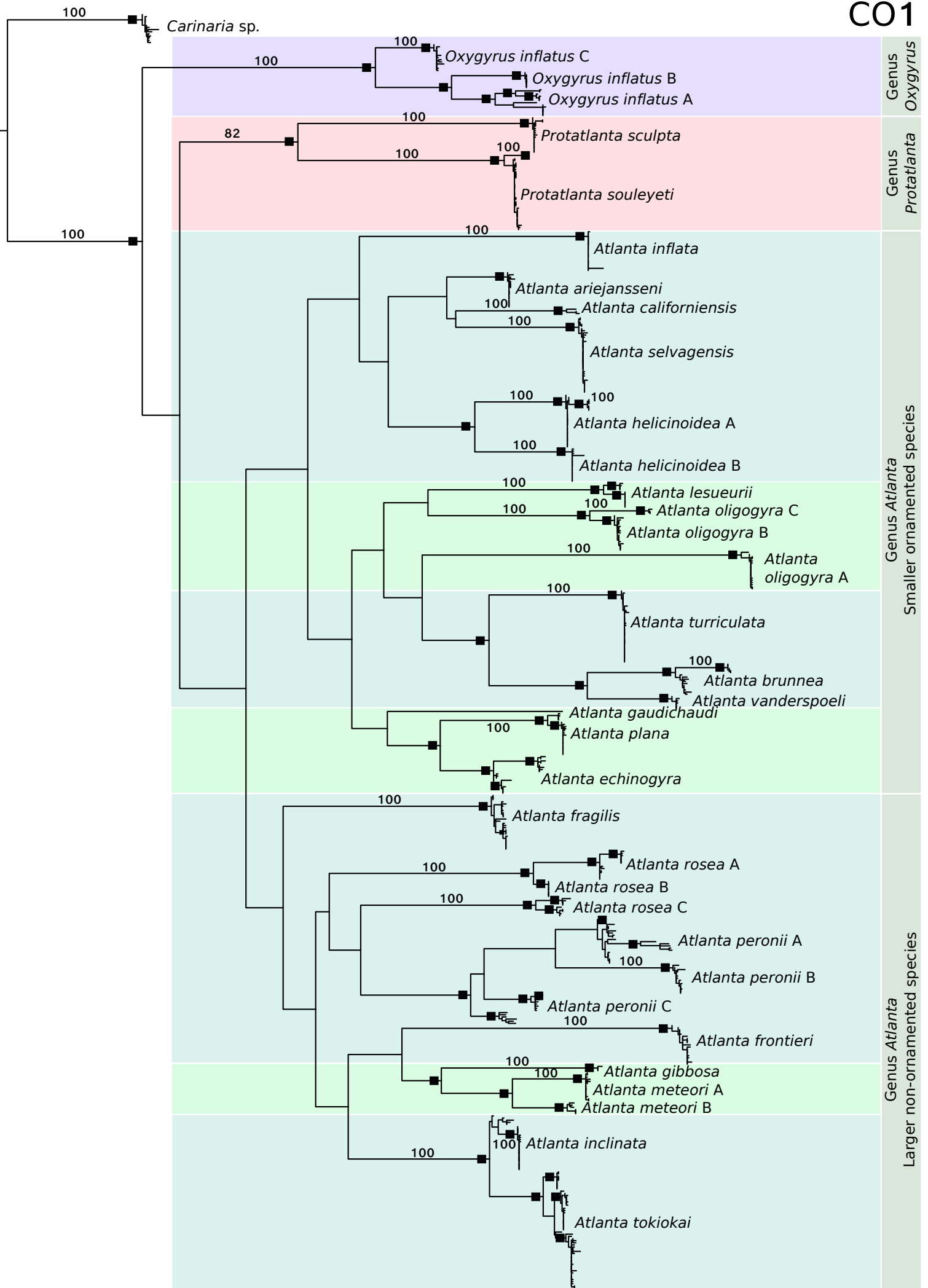

Supplement: Supplementary file 2 — Additional file 2: Supplementary Figure 1. Maximum likelihood phylogeny of the family Atlantidae based on cytochrome c oxidase subunit 1 mitochondrial DNA (CO1). Black squares represent bootstrap support > 80%. Species groups based on morphology are highlighted with coloured boxes (See Table 1). [file 12862_2020_1682_MOESM2_ESM.pdf]

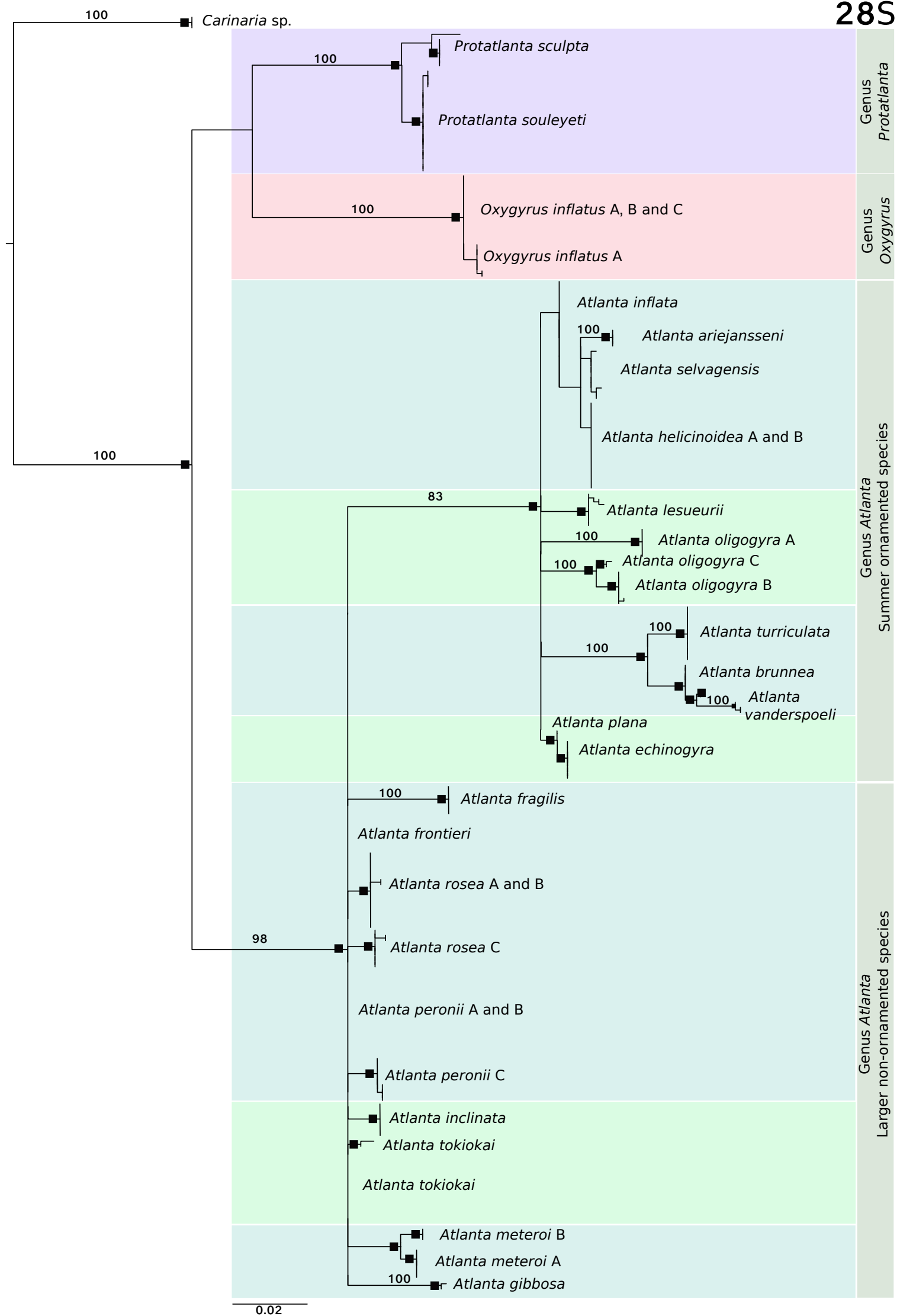

Supplement: Supplementary file 3 — Additional file 3: Supplementary Figure 2. Maximum likelihood phylogeny of the family Atlantidae based on the nuclear gene 28S. Poorly supported branches (< 60%) have been collapsed to simplify the phylogeny. Black squares represent bootstrap support > 80%. Species groups based on morphology are highlighted with coloured boxes (See Table 1). [file 12862_2020_1682_MOESM3_ESM.pdf]

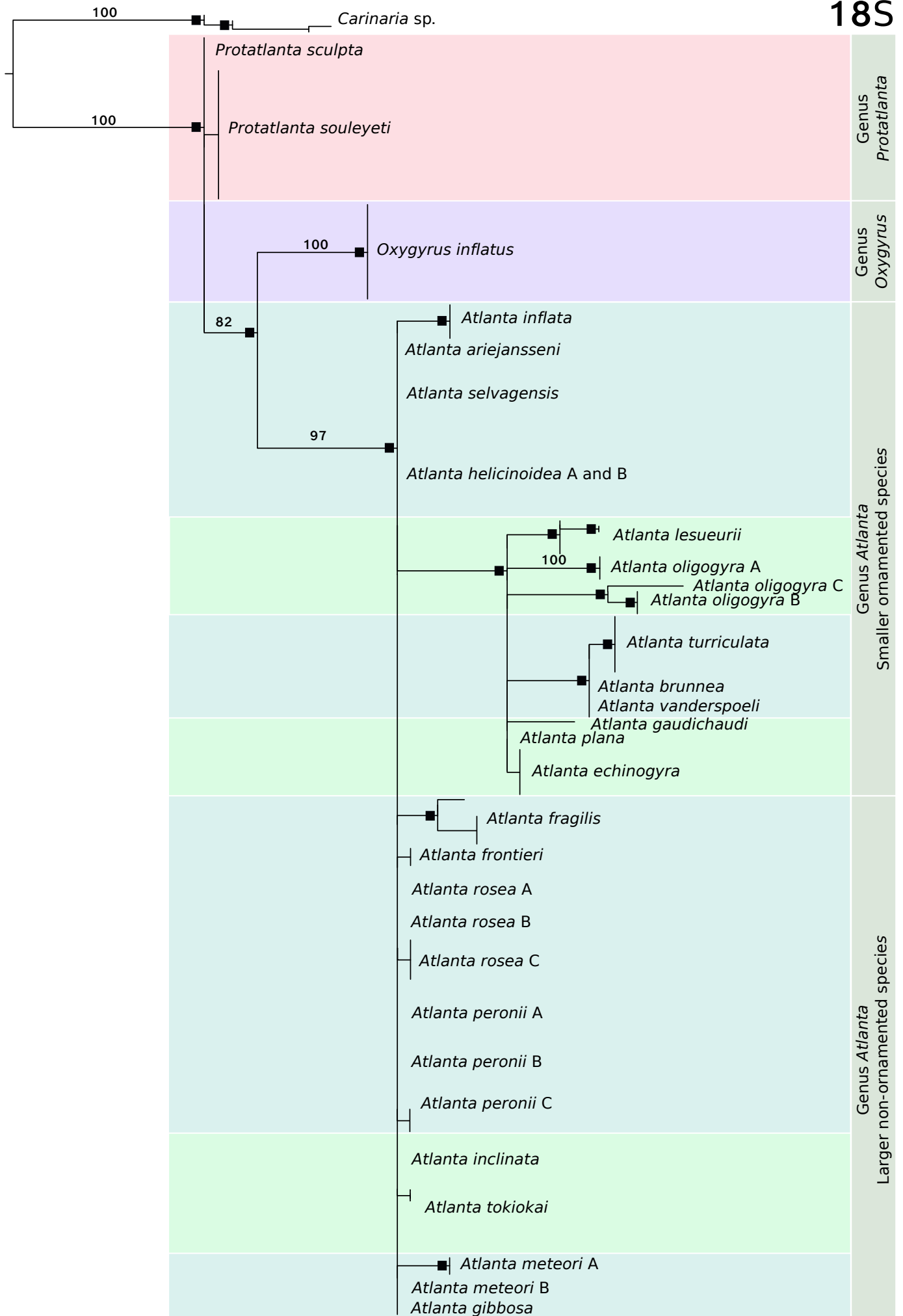

Supplement: Supplementary file 4 — Additional file 4: Supplementary Figure 3. Maximum likelihood phylogeny of the family Atlantidae based on the nuclear gene 18S. Poorly supported branches (< 60%) have been collapsed to simplify the phylogeny. Black squares represent bootstrap support > 80%. Species groups based on morphology are highlighted with coloured boxes (See Table 1). [file 12862_2020_1682_MOESM4_ESM.pdf]

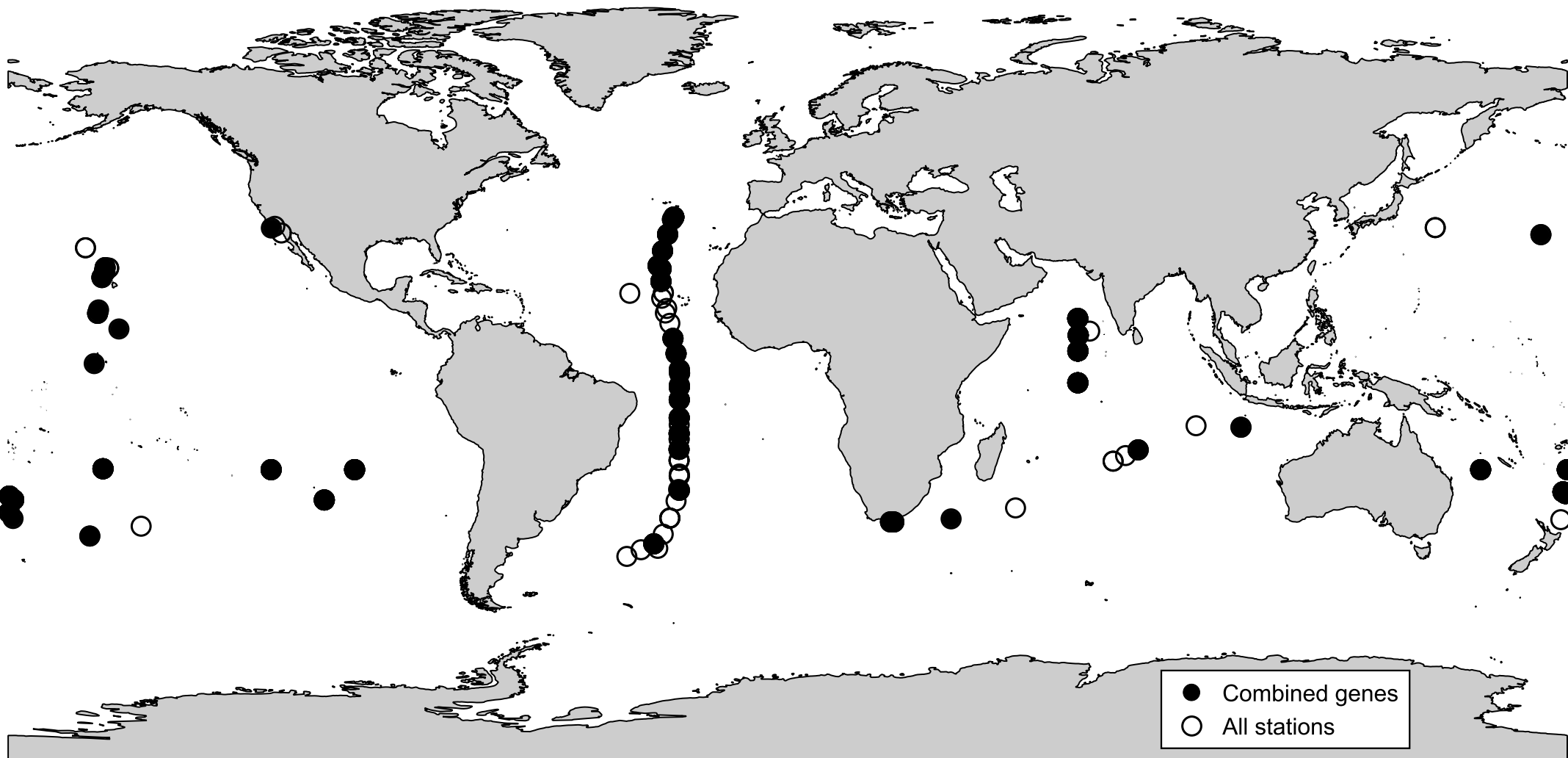

Supplement: Supplementary file 5 — Additional file 5 : Supplementary Figure 4. The distribution of all specimens used in this study demonstrates the global coverage of the dataset. Filled circles represent specimens used for the concatenated gene phylogeny. Data were visualised using the software QGIS v2.8 (https://www.qgis.org/en/site/). [file 12862_2020_1682_MOESM5_ESM.pdf]
